# Evidence of Selection against Complex Mitotic-Origin Aneuploidy during Preimplantation Development

**DOI:** 10.1371/journal.pgen.1005601

**Published:** 2015-10-22

**Authors:** Rajiv C. McCoy, Zachary P. Demko, Allison Ryan, Milena Banjevic, Matthew Hill, Styrmir Sigurjonsson, Matthew Rabinowitz, Dmitri A. Petrov

**Affiliations:** 1 Department of Biology, Stanford University, Stanford, California, United States of America; 2 Natera, Inc., San Carlos, California, United States of America; University of Southern California, UNITED STATES

## Abstract

Whole-chromosome imbalances affect over half of early human embryos and are the leading cause of pregnancy loss. While these errors frequently arise in oocyte meiosis, many such whole-chromosome abnormalities affecting cleavage-stage embryos are the result of chromosome missegregation occurring during the initial mitotic cell divisions. The first wave of zygotic genome activation at the 4–8 cell stage results in the arrest of a large proportion of embryos, the vast majority of which contain whole-chromosome abnormalities. Thus, the full spectrum of meiotic and mitotic errors can only be detected by sampling after the initial cell divisions, but prior to this selective filter. Here, we apply 24-chromosome preimplantation genetic screening (PGS) to 28,052 single-cell day-3 blastomere biopsies and 18,387 multi-cell day-5 trophectoderm biopsies from 6,366 *in vitro* fertilization (IVF) cycles. We precisely characterize the rates and patterns of whole-chromosome abnormalities at each developmental stage and distinguish errors of meiotic and mitotic origin without embryo disaggregation, based on informative chromosomal signatures. We show that mitotic errors frequently involve multiple chromosome losses that are not biased toward maternal or paternal homologs. This outcome is characteristic of spindle abnormalities and chaotic cell division detected in previous studies. In contrast to meiotic errors, our data also show that mitotic errors are not significantly associated with maternal age. PGS patients referred due to previous IVF failure had elevated rates of mitotic error, while patients referred due to recurrent pregnancy loss had elevated rates of meiotic error, controlling for maternal age. These results support the conclusion that mitotic error is the predominant mechanism contributing to pregnancy losses occurring prior to blastocyst formation. This high-resolution view of the full spectrum of whole-chromosome abnormalities affecting early embryos provides insight into the cytogenetic mechanisms underlying their formation and the consequences for human fertility.

## Author Summary

By day 3 of development, more than half of human embryos contain at least one cell that deviates from the typical 46-chromosome complement. These whole-chromosome abnormalities include polyploidies, which affect the entire chromosome set, as well as aneuploidies, which involve gains and losses of particular chromosomes. The rate of aneuploidy increases with maternal age, primarily due to chromosome segregation errors arising during egg formation (maternal meiosis). While some forms of aneuploidy, such as Trisomy 21, are compatible with live birth, most aneuploid embryos do not survive to term. Our study applied genetic techniques to screen early embryos from *in vitro* fertilization cycles, demonstrating that while diverse whole-chromosome abnormalities can be observed at early developmental stages, these errors are strongly filtered during preimplantation development. Specifically, errors occurring during the initial post-fertilization cell divisions often result in the simultaneous loss of multiple chromosomes, a pattern consistent with abnormal cell division. Our data provide evidence of selection against this class of aneuploidy before day 5 of development, thus reducing fertility. Patients referred for genetic screening due to previous IVF failure had higher rates of mitotic error, highlighting its clinical relevance and indicating that patient-specific genetic and environmental factors influence error rates.

## Introduction

Human reproduction is inefficient, with pregnancy loss estimated to occur in approximately 70% of all conceptions [1]. The majority of pregnancy losses take place before 12 weeks of gestation [2] and are mostly explained by whole-chromosome abnormalities [3, 4] as well as structural aberrations [5]. Our study focuses on numerical abnormalities affecting whole chromosomes, the detection of which has been extensively validated [6]. These errors can be broadly classified as polyploidy (non-diploid multiples of the haploid chromosome set) and aneuploidy (other configurations of extra or missing chromosomes). Given the strong implications for fertility, a clear understanding of the rates and molecular mechanisms contributing to various classes of whole-chromosome abnormalities is an important goal in reproductive medicine and human biology in general.

It has long been established that incidence of aneuploidy affecting maternal chromosome copies increases with maternal age [7]. This pattern is driven mostly by errors occurring during maternal meiosis, which arrests at the diplotene stage until it resumes at ovulation many years later [3]. These meiotic errors were at first thought to arise primarily via whole-chromosome non-disjunction—the failure of homologous chromosomes or sister chromatids to separate [3]. Later work, however, demonstrated a greater role of unbalanced predivision—the premature separation and subsequent missegregation of sister chromatids [8]—in contributing to maternal age-related meiotic error and implicated breakdown of cohesin proteins as a possible mechanism [9–11]. Both non-disjunction and unbalanced chromatid predivision result in a chromosome gain in one daughter cell with a corresponding chromosome loss in the other daughter cell, but can be distinguished when both oocytes or embryos and their corresponding polar bodies are analyzed. A recent study used this approach to confirm the preponderance of unbalanced chromatid predivision and also identify a non-canonical segregation pattern whereby sister chromatids separate at the meiosis I (MI), followed by non-random segregation at meiosis II (MII) favoring separation of homologous chromosomes [12].

In addition to meiotic errors, mitotic errors are extremely common during the initial post-zygotic cell divisions and produce mosaic embryos containing multiple distinct karyotypes [13]. It has been estimated that a large proportion of embryos tested during *in vitro* fertilization (IVF) are mosaics [6, 14–18], though the incidence of mosaicism varies widely depending on embryonic stage investigated and method of analysis [13]. This high rate of mitotic error is presumably due to relaxed cell cycle control during the initial embryonic cell divisions, but which is reestablished prior to blastocyst formation [19]. While a subset of mosaic embryos may survive due to self-correction [20], a substantial proportion of mosaic embryos are inviable and arrest before the blastocyst stage [21–23]. Despite these apparent consequences for embryonic survival, rates of mitotic error are yet to be reported for individual chromosomes based on a large survey of early embryos.

The greatest source of information about chromosome abnormalities is preimplantation genetic screening (PGS) conducted during IVF, whereby cells are biopsied from day-3 or day-5 embryos and the copy number of one or more chromosomes is determined. Embryos that test euploid are then recommended for transfer to improve the rate of implantation and live birth in IVF. While early versions of PGS employed fluorescence *in situ* hybridization (FISH) and were thus limited to testing only a few chromosomes at a time, microarray-based approaches are capable of assaying ploidy status of all chromosomes simultaneously. One powerful approach, termed array comparative genomic hybridization (aCGH), measures copy number aberrations by contrasting relative signal intensities of test and reference samples on a DNA microarray [24]. By combining this approach with single nucleotide polymorphism (SNP) profiling, more recent microarray-based technologies can differentiate between maternal and paternal homologs, thus shedding additional light on the parental origin and timing of chromosome missegregation.

A recent study applied 24-chromosome SNP-microarray PGS to 15,169 trophectoderm (TE) biopsies from day-5 embryos, documenting ploidy at the blastocyst stage with extreme precision [25]. Our data demonstrate, however, that day-5 embryos contain a biased subset of whole-chromosome abnormalities that have already been filtered by self-correction and selection at the onset of zygotic genome activation at the 4–8 cell stage. In fact, embryonic arrest before day 5 can be responsible for the loss of more than half of IVF embryos, the vast majority of which are non-euploid [26]. Indeed, screening of blastocyst biopsies is preferable in the context of IVF, in part because survival to day 5 is an indicator of developmental competence [27]. Thus, the full spectrum of whole-chromosome abnormalities is only observable at earlier developmental time points, motivating a comparably large study of cleavage-stage embryos with 24-chromosome SNP-based PGS.

Here, we present 24-chromosome PGS results from 28,052 individual day-3 blastomeres and 18,387 multi-cell day-5 TE biopsies collected from a total of 6,366 IVF cycles, characterizing frequencies of both common and rare ploidy states and contrasting those detected at each sampling point. These data suggest that embryos purged in early development often experienced catastrophic mitotic errors, while meiotic errors—which tend to result in minor aneuploidy or polyploidy—are comparatively viable through blastocyst formation. Consistent with this interpretation, we show that patients referred for PGS due to repeat IVF failure had higher rates of mitotic error than patients with other clinical indications, suggesting that some of these patients suffer systematically higher rates of preclinical pregnancy loss due to mitotic aberrations. By inferring ploidy status of all chromosome pairs and distinguishing parental origin of the affected homologs, we achieve a high-resolution view of whole-chromosome abnormalities that provides key insights into the characteristics of chromosome segregation and the impacts on early development.

## Results

### Patient demographics and sample description

**Fig 1:**
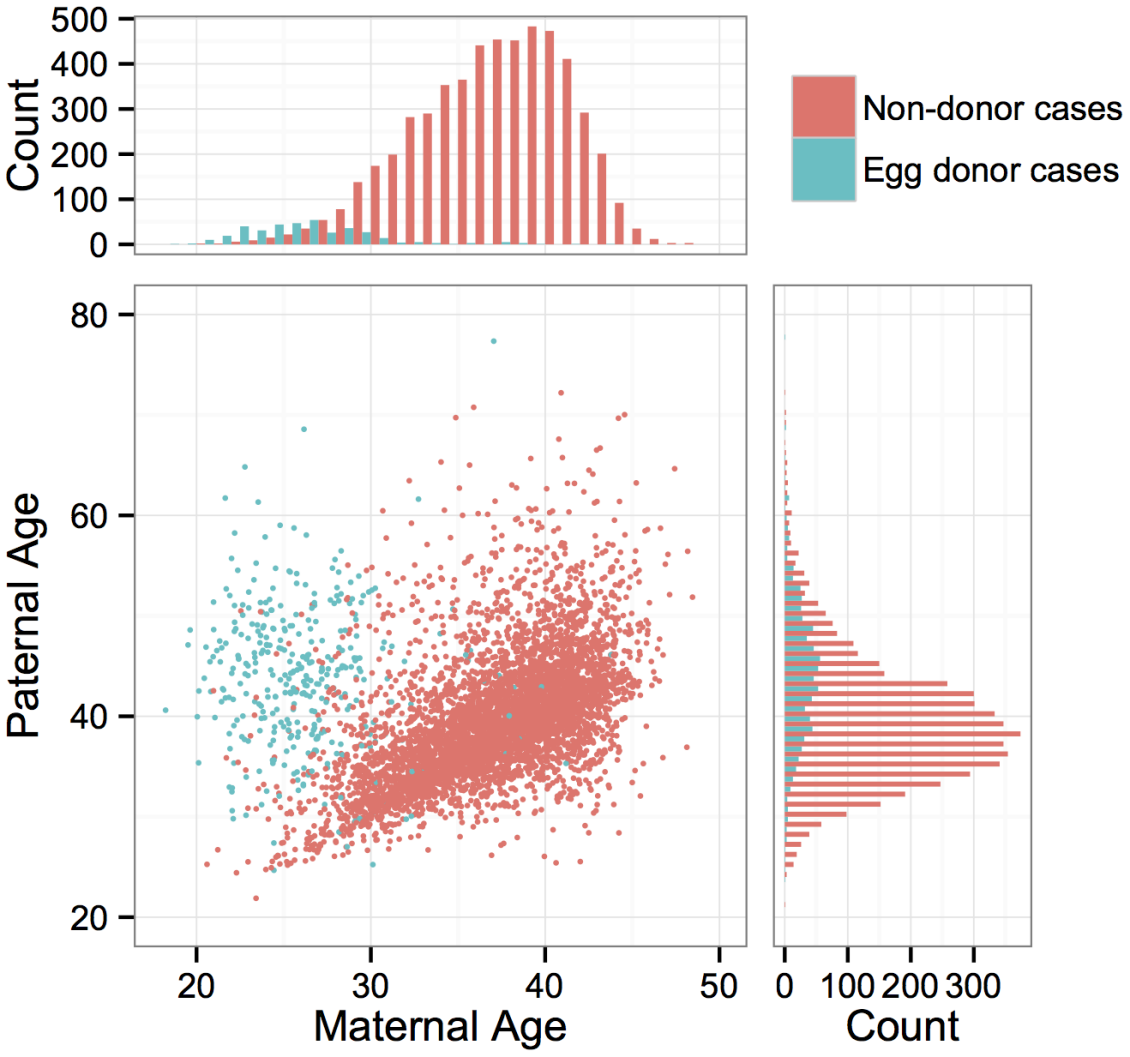
Distributions of maternal and paternal ages and correlation between parents’ ages. Colors indicate whether the maternal age refers to an egg donor or a non-donor patient.

**Fig 2:**
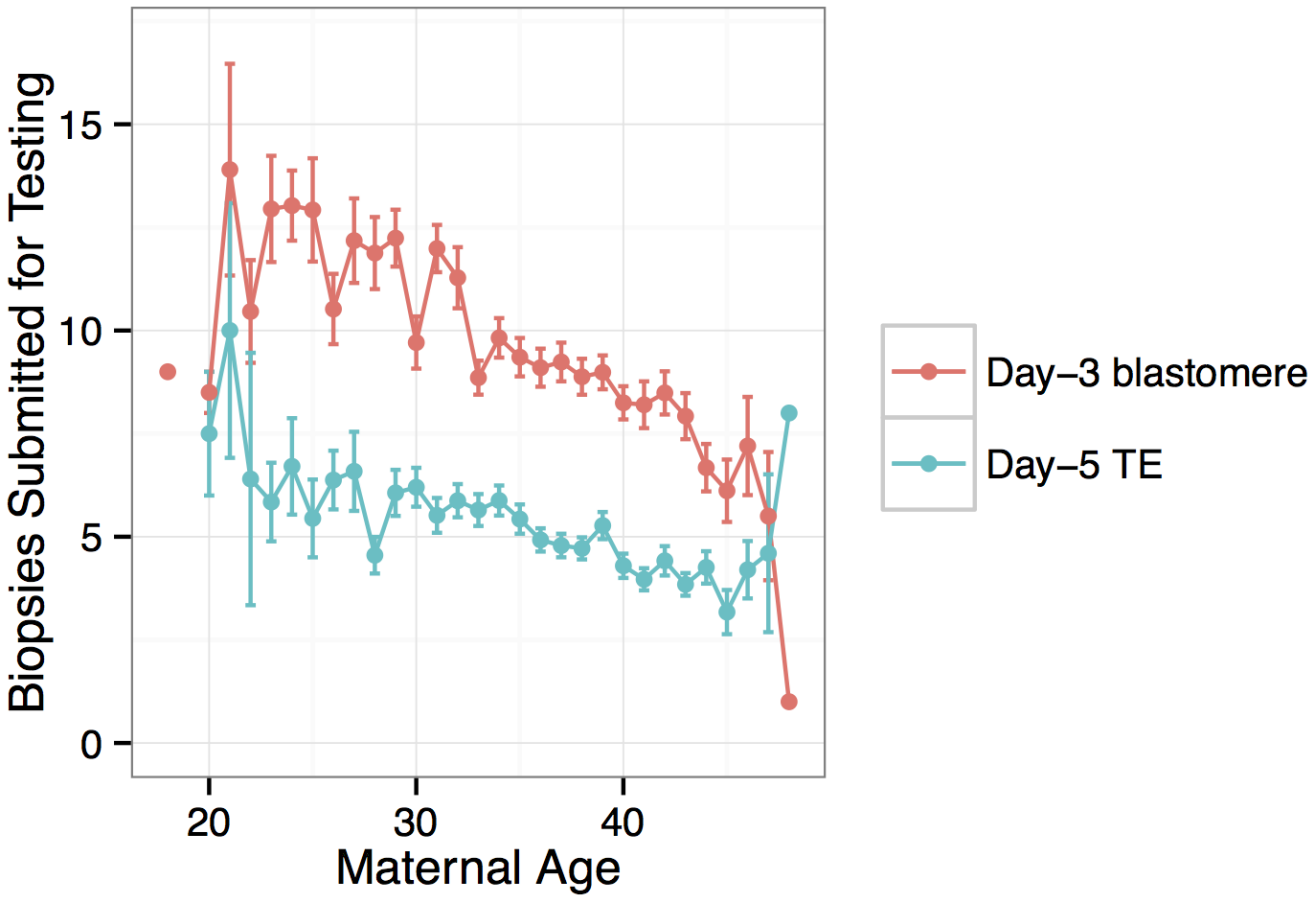
Number of embryo biopsies submitted for PGS declines with maternal age for both day-3 blastomere biopsies and day-5 TE biopsies. Error bars indicate standard errors of the means.

PGS was conducted for a total of 6,366 anonymous IVF cases referred to Natera between February 2009 and March 2014 by a total of 181 IVF centers. The mean maternal age was 36.3 (including egg donors) and the mean paternal age was 40.3 (Fig 1). Of these cases, a total of 890 cases utilized egg donors, among whom the mean age was 26.3 (Fig 1). The data are composed of PGS results from 28,052 individual day-3 blastomeres and 18,387 5–10 cell day-5 TE biopsies. Patients submitted means of 9.6 blastomere biopsies or 5.1 TE biopsies per case, with the number of submitted samples of both types declining with increasing maternal age (day-3 blastomeres: *β* = −0.0232, *SE* = 0.00203, *P* < 1 × 10^−10^; day-5 TE biopsies: *β* = −0.0257, *SE* = 0.00251, *P* < 1 × 10^−10^; Fig 2). A total of 3,767 cases (59.2%) reported the reason for their referral, with advanced maternal age, recurrent pregnancy loss, and gender selection constituting the most common indications (Table 1).

**Table 1:** Reported reason for referral for PGS of IVF embryos. A total of 3,767 of 6,366 cases reported the reason for referral. The table reports referral reasons for cases submitting at least one day-3 blastomere biopsy or one day-5 TE biopsy, respectively, as well as the total number of cases. Row sums exceed the row totals because some cases provided both sample types. Column sums exceed the grand total of 3,767 because some cases provided multiple reasons for referral.

Referral reason	Day-3 blastomere	Day-5 TE	Total cases
Advanced maternal age	365	1195	1554
Repeat pregnancy loss	257	665	920
Gender selection	513	339	838
Previous IVF failure	179	408	584
Male factor	70	276	345
Unexplained infertility	65	276	341
Previous aneuploidy	105	219	320
Translocation	48	90	138
*No data*	1674	1034	2599

For 40 cases, no reliable ploidy calls could be made for any embryo, limiting our analysis to the remaining 6,326 cycles. Of 46,449 total samples, DNA was not detected for 2,653 samples (5.7%). These samples were therefore excluded from all subsequent analyses. An additional 1,071 samples had low-confidence calls (< 80% confidence) for greater than four chromosomes and were also excluded for quality-control purposes. All other low-confidence calls were masked and considered as missing data. At least one error affecting a whole chromosome was detected for total of 15,842 (62.1%) of the remaining 25,497 blastomeres as compared to 7,623 (44.3%) of 17,219 TE biopsies, a highly significant difference [*χ* ^2^(1, *N* = 42,716) = 1323.8, *P* < 1 × 10^−10^]. For an additional 323 blastomeres and 146 TE biopsies, only segmental deletions or duplications were detected. We note that in the face of mosaicism, which is common in cleavage-stage embryos, ploidy of individual blastomeres will not necessarily reflect the ploidy status of the entire embryo, but rather provides a snapshot of a single cell at this early developmental stage. An additional caveat of our analysis is that mosaicism within TE biopsies may not be detectable if a small proportion of cells are affected. Ploidy inference was conducted using the Parental Support algorithm [6], which does not explicitly test for mosaicism, but uses a Bayesian methodology to calculate likelihoods of ploidy hypotheses (assuming the absence of mosaicism) given the data. This limitation must therefore be considered when comparing results between sample types.

### Association of whole-chromosome abnormalities with parental ages

**Fig 3:**
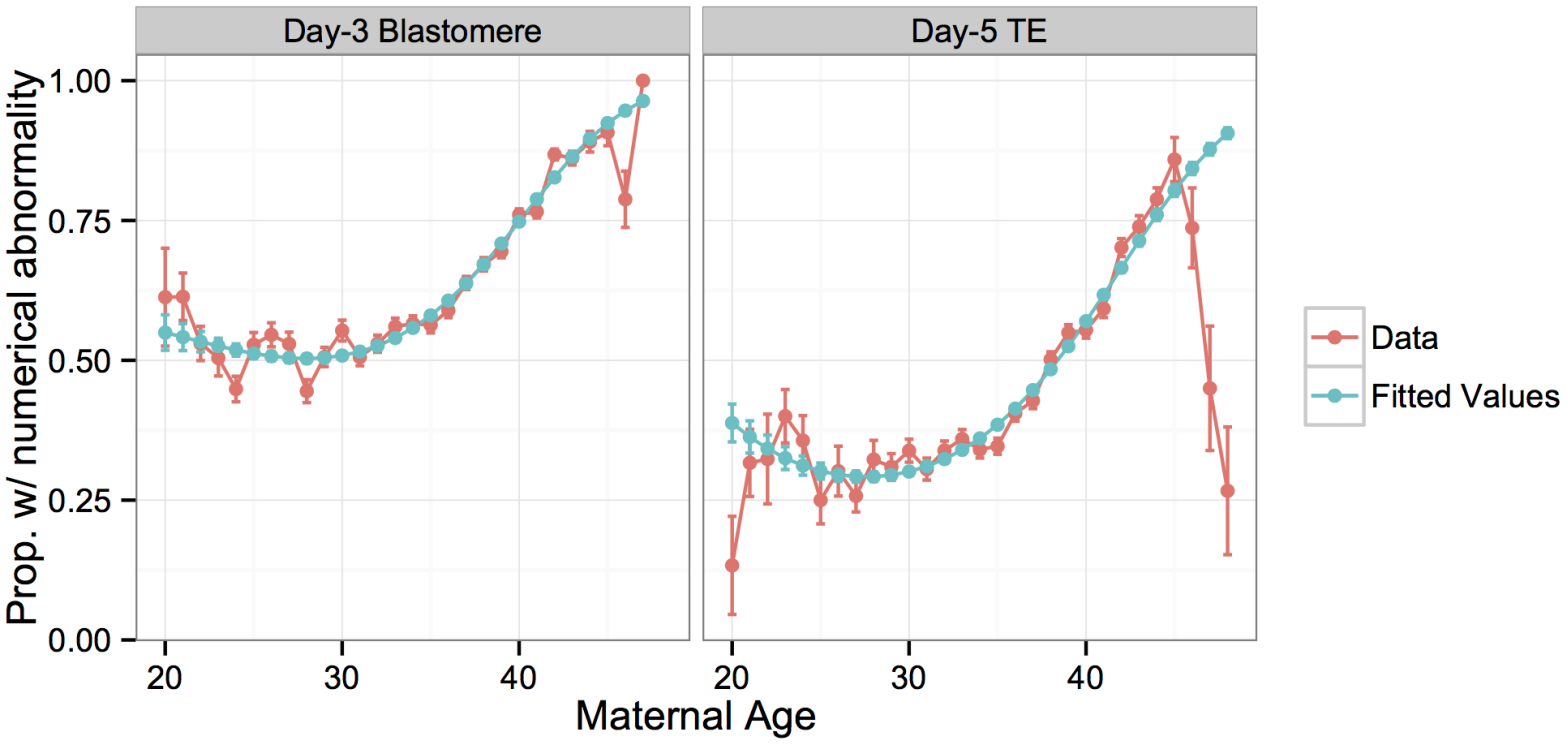
Rate of whole-chromosome abnormalities versus maternal age compared to fitted values from a logistic regression model. Errors bars for the data indicate standard errors of the proportions, while error bars for the model indicate standard errors of the fitted means.

Many studies have demonstrated that the incidence of aneuploidy increases with maternal age, starting in the mid-thirties, driven primarily by errors of maternal meiotic origin [3, 28, 29]. Our data replicate these results, with a significant increase in per-case proportion of samples with whole-chromosome abnormalities with increasing maternal age (Fig 3). This relationship is best fit by a third-order polynomial model in the case of the blastomere biopsies (S1 Table; McFadden’s pseudo-*R* ^2^ = 0.270), and a second-order polynomial in the case of the TE biopsies (S1 Table; McFadden’s pseudo-*R* ^2^ = 0.189). The model provided poor fit for TE biopsy patients in the upper tail of the age distribution, who had strikingly lower rates of whole-chromosome abnormalities than expected. While the sample size is limited in this upper tail, the consistently lower rates of abnormalities in day-5 embryos from mothers > 45 years old suggest a potential selection bias. The small subset of patients in this age group who are capable of producing embryos surviving to day 5 may have systematically lower rates of meiotic or mitotic error, a hypothesis that merits future investigation.

Given the well-established association between fertility and embryonic euploidy, it should be noted that specific rates of meiotic and mitotic error reported in this study are likely particular to the IVF population. Previous studies also demonstrated that ovarian stimulation and IVF culture conditions can both influence rates of chromosome abnormalities [30]. Nevertheless, high rates of meiotic and mitotic error have been observed even for unstimulated cycles and for patients without obstetrical or gynecological pathologies [31, 32], suggesting that basic insights provided by this study can be generalized to better understand natural human fertility. In support of this conclusion, we detected no significant difference in rates of whole-chromosome abnormalities among day-3 blastomeres [*F*(1,2647) = 1.693, *P* = 0.193] or day-5 TE biopsies [*F*(1,3165) = 0.615, *P* = 0.433] in embryos from fertile egg donors and non-donor IVF patients after accounting for maternal age effects (S2B Fig).

The pattern of association between whole-chromosome abnormalities and maternal age was strongly chromosome-specific (S1 Fig), as has been reported based on smaller samples from different developmental time points [33]. The maternal age effect on aneuploidy is considered the primary reason for the corresponding age-associated decline in female fertility, both in the contexts of natural conception and IVF. Our data are consistent with this interpretation, as the relationship between maternal age and the rate of whole-chromosome abnormalities closely mirrored the relationship between maternal age and various measures of IVF success in public data obtained from the 2011 CDC National Summary Report [34] (S2A Fig).

The question of whether risk for whole-chromosome abnormalities is affected by paternal age is contentious and is complicated by the fact that maternal and paternal ages are often highly correlated [35]. This was also the case in our study, with strong correlation between parental ages (Fig 1; *r* = 0.334, *P* < 1 × 10^−10^), especially when egg donors were excluded (Fig 1; *r* = 0.536, *P* < 1 × 10^−10^) driving a strong relationship between whole-chromosome abnormalities and paternal age in blastomeres (*β* = 0.0268, *SE* = 0.00288, *P* < 1 × 10^−10^) and TE biopsies (*β* = 0.0449, *SE* = 0.00342, *P* < 1 × 10^−10^). When limiting analysis to egg donor cases, among which maternal and paternal ages were not correlated (*r* = −0.0672, *P* = 0.204), a marginal association was still detected between the rate of whole-chromosome abnormalities and paternal age in day-3 blastomeres (*β* = 0.0123, *SE* = 0.00612, *P* = 0.0456), but not in day-5 TE biopsies (*β* = −0.0119, *SE* = 0.0181, *P* = 0.514).

Intrigued by the potential effect of paternal age, we employed complementary statistical approaches to control for maternal age and test for a residual paternal age effect on risk for whole-chromosome abnormalities. Spearman partial correlation detected a significant association between paternal age and proportion of affected samples upon holding maternal age constant for both variables in day-3 blastomeres (*r*_*xy*.*z*_ = 0.0613, *P* = 0.00191), but not in day-5 TE biopsies (*r*_*xy*.*z*_ = 0.0167, *P* = 0.362). As expected, the same approach applied to maternal age upon holding paternal age constant detected a much stronger association in both sample types (day-3 blastomeres: *r*_*xz*.*y*_ = 0.430, *P* < 1 × 10^−10^; day-5 TE biopsies: *r*_*xz*.*y*_ = 0.302, *P* < 1 × 10^−10^).

Using an alternative approach, we stratified the sample into two groups: fathers younger than the median paternal age (39.6 for blastomere cases and 38.9 for TE biopsy cases) and fathers equal to or older than the median paternal age. We then matched cases sampled from each paternal age group on maternal age (within 0.1 standard deviations of the maternal age), dropping unmatched cases and randomly breaking ties. Controlling for maternal age in this manner, we found that increased paternal age was again marginally associated with an increased rate of whole-chromosome errors in day-3 blastomere biopsies [*χ* ^2^(1, *N* = 12,263) = 4.980, *P* = 0.0256] but not in day-5 TE biopsies [*χ* ^2^(1, *N* = 8055) = 0.028, *P* = 0.857]. While significant, the blastomere biopsy effect was small, with 61.7% affected blastomeres for fathers less than 39.6 years old versus 63.9% for fathers greater than or equal to 39.6 years old.

In contrast, a logistic GLM including paternal age as a predictor variable did not provide significantly better fit than a reduced model that only accounted for maternal age for either day-3 blastomere biopsies [*F*(1,2549) = 2.169, *P* = 0.141] or day-5 TE biopsies [*F*(1,2970) = 0.994, *P* = 0.319].

### Diverse whole-chromosome abnormalities detected at days 3 and 5

**Fig 4:**
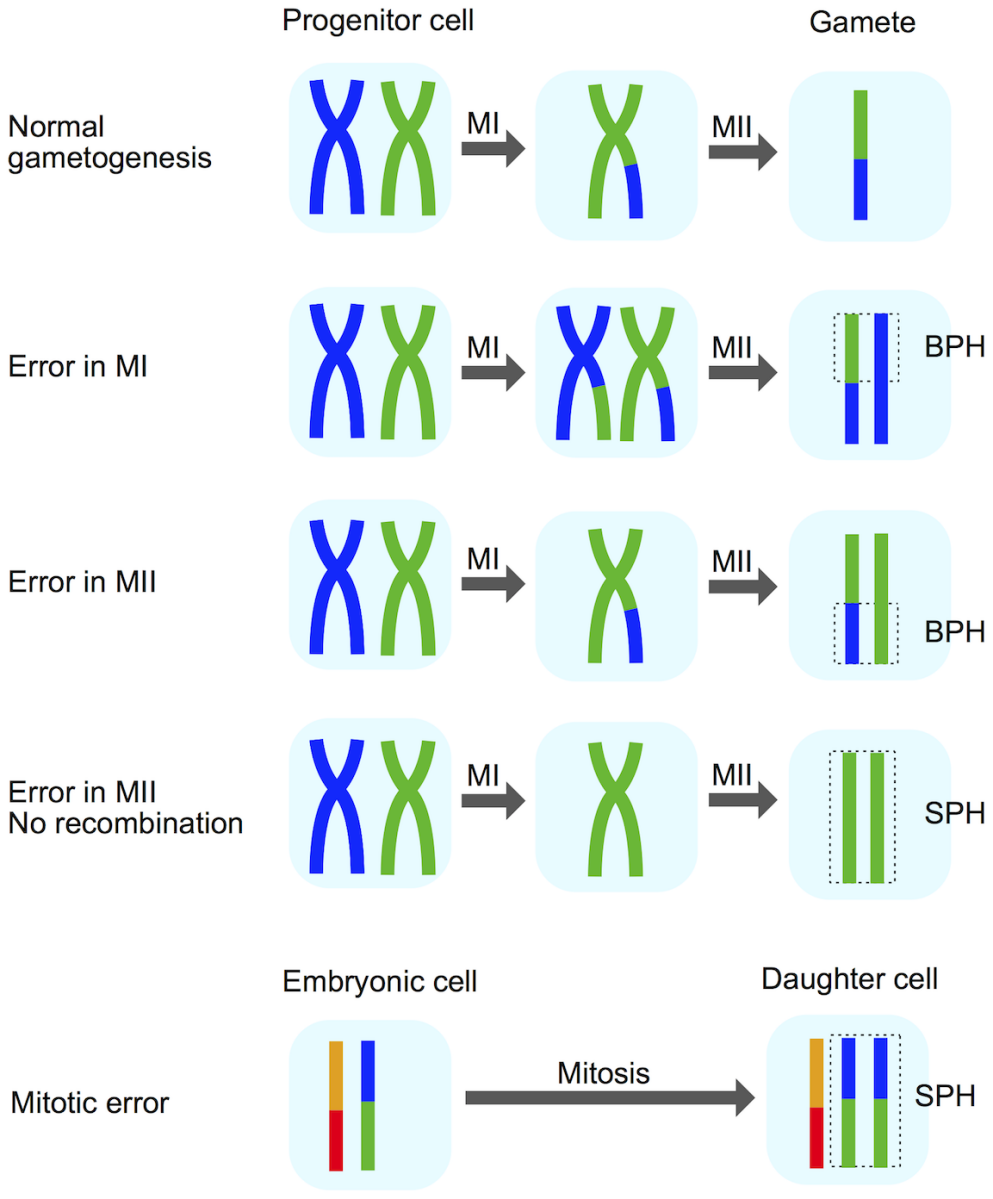
Schematic explaining the BPH signature of meiotic-origin chromosome gain. Certain chromosomal signatures are indicative of meiotic versus mitotic origin of aneuploidy formation. The presence of three unmatched haplotypes (‘both parental homologs’; BPH) in any chromosomal region of the embryo suggests an error in either meiosis I (MI) or meiosis II (MII). Chromosome gains involving identical homologs (‘single parental homolog’; SPH) can arise either by mitotic error or MII errors in the absence of recombination [6, 36]. Reprinted from [36] with permission from Elsevier.

We next sought to stratify different forms of whole-chromosome abnormalities to better understand the mechanisms underlying their formation. Certain chromosomal signatures are strongly indicative of either meiotic or mitotic error, while other signatures can arise via either process. One signature of chromosome gains that can help identify meiotic error is the presence of three unmatched haplotypes in a given region of the embryo’s genome (i.e. two non-identical, but homologous chromosomes inherited from a single parent; Fig 4). This signature, which we termed ‘both parental homologs’ (BPH) error, is unique to meiosis, and previous literature suggests that it primarily arises due to unbalanced chromatid predivision [8, 10]. Mitotic errors, as well as meiotic errors in the absence of recombination, produce chromosome gains in which the extra chromosome is identical to another chromosome over its entire length (Fig 4). We refer to these chromosome gains as ‘single parental homolog’ (SPH) errors. This logic was first introduced by Johnson et al. [6] (also see [36]) and was recently employed to distinguish meiotic-origin aneuploidies for a genome wide association study of aneuploidy risk [37].

**Fig 5:**
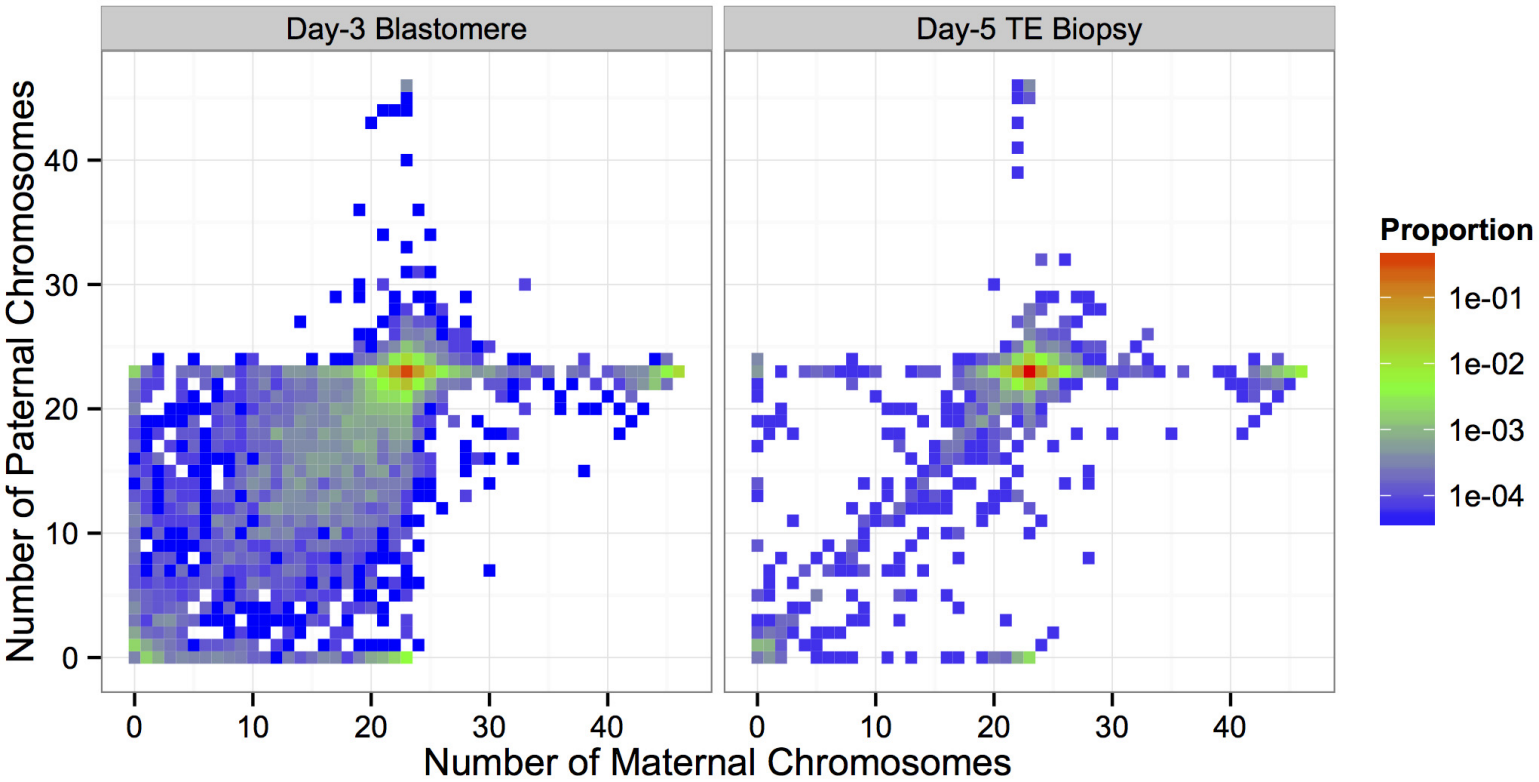
Heat maps depicted proportions of biopsies with different configurations of maternal and paternal chromosomes. Minor aneuploidies and triploidies disproportionately involved maternal homologs. The paternal genome is absent from the majority of haploid embryos. Sub-diploid complements with missing maternal and paternal homologs were common in day-3 blastomere biopsies, but strongly depleted in day-5 TE biopsies. This results in a ‘steeper’ heat map surface for day-5 biopsies, centered on the euploid complement.

We tabulated the total counts of different forms of whole-chromosome abnormalities, contrasting those observed at day 3 and day 5 (Table 2; Fig 5). We found that errors affecting few chromosomes (single trisomies, single monosomies) were biased in their impact on maternal homologs, supporting a maternal meiotic origin of formation. Consistent with this interpretation, more than half of maternal trisomies carried the BPH signature in both day-3 blastomeres and day-5 TE biopsies (Table 2). Meanwhile, complex aneuploidies involving multiple chromosomes were approximately balanced in their effect on maternal and paternal homologs (Fig 5), suggesting that the mechanism underlying these errors is primarily post-zygotic and does not discriminate based on parental origin. Errors involving multiple chromosomes were strongly biased toward chromosome losses over chromosome gains, and were largely depleted by day 5 of development (Table 2; Fig 5).

**Table 2:** Rates of various forms of whole-chromosome abnormalities observed in day-3 blastomere biopsies and day-5 TE biopsies. Counts and proportions of total sample are reported for each sample type. Complex errors involving multiple chromosomes decrease in frequency between days 3 and 5, while errors of putative meiotic origin (e.g. maternal BPH trisomy) display a corresponding increase. Maternal triploidies are defined as containing an extra set of maternal chromosomes. Maternal haploidies are defined as containing only a maternal set, but no paternal set of chromosomes. Paternal triploidies and haploidies follow this same naming convention with respect to paternal chromosome sets. Near-triploidies and near-haploidies are arbitrarily defined as having 20+ extra or missing chromosomes, respectively.

Class of whole-chromosome abnormality	*N* blastomeres (% ± SE)	*N* TE biopsies (% ± SE)
Minor aneuploidies (≤ 2 chromosomes affected)		
Single trisomy	2013 (7.90 ± 0.17%)	1927 (11.19 ± 0.24%)
Single maternal trisomy	1695 (6.65 ± 0.16%)	1606 (9.33 ± 0.22%)
Single maternal BPH trisomy	1164 (4.57 ± 0.13%)	1031 (5.99 ± 0.18%)
Single maternal SPH trisomy	531 (2.08 ± 0.09%)	575 (3.34 ± 0.14%)
Single paternal trisomy	318 (1.25 ± 0.07%)	321 (1.86 ± 0.10%)
Single paternal BPH trisomy	41 (0.16 ± 0.03%)	45 (0.26 ± 0.04%)
Single paternal SPH trisomy	277 (1.11 ± 0.06%)	276 (1.60 ± 0.10%)
Single monosomy	2720 (10.67 ± 0.19%)	1838 (10.67 ± 0.24%)
Single maternal monosomy	2088 (8.19 ± 0.17%)	1565 (9.09 ± 0.22%)
Single paternal monosomy	632 (2.48 ± 0.10%)	273 (1.59 ± 0.10%)
Single nullisomy	153 (0.60 ± 0.05%)	34 (0.20 ± 0.03%)
Double error	2334 (9.15 ± 0.18%)	1376 (7.99 ± 0.21%)
Errors of ploidy (20+ chromosomes affected)		
Triploidy / near-triploidy	751 (2.95 ± 0.11%)	295 (1.71 ± 0.10%)
Maternal (digynic) triploidy / near-triploidy	725 (2.84 ± 0.10%)	271 (1.57 ± 0.09%)
Paternal (diandric) triploidy / near-triploidy	23 (0.09 ± 0.02%)	22 (0.13 ± 0.03%)
Haploidy / near-haploidy	306 (1.20 ± 0.07%)	98 (0.57 ± 0.06%)
Maternal haploidy / near-haploidy	228 (0.89 ± 0.06%)	80 (0.46 ± 0.05%)
Paternal haploidy / near-haploidy	72 (0.28 ± 0.03%)	18 (0.10 ± 0.02%)
Complex errors		
3–19 chromosomes affected	6304 (24.72 ± 0.27%)	1790 (10.40 ± 0.23%)

Triploidy (and near-triploidy) primarily affected maternal homologs (Table 2; Fig 5). We note that this excess of maternal (digynic) triploidy compared to paternal (diandric) triploidy is at odds with some earlier literature [38]. This discrepancy is explained, however, by the current widespread use of intracytoplasmic sperm injection (ICSI) in place of conventional IVF [39]. While most cases of triploidy in conventional IVF are due to dispermic fertilization and produce diandric embryos, these errors are essentially eliminated by ICSI, while the rate of digynic tripronuclear (3PN) zygote formation is estimated at 2.5–6.2% [40]. These 3PN zygotes are formed when a diploid oocyte—arising via meiotic error—is fertilized by a haploid sperm [41]. The relative occurrences of digynic and diandric triploidies in our data (Table 2) are therefore consistent with the literature, as 80–90% of IVF cases analyzed by Natera utilized ICSI. Haploid (and near-haploid) embryos possessing only the maternally inherited genome greatly exceeded those possessing only the paternally inherited genome (Table 2; Fig 5). This is also consistent with previous data from ICSI and is thought to arise primarily due to gynogenesis, whereby the oocyte is stimulated by a sperm factor, but the sperm chromatin fails to decondense [42].

**Fig 6:**
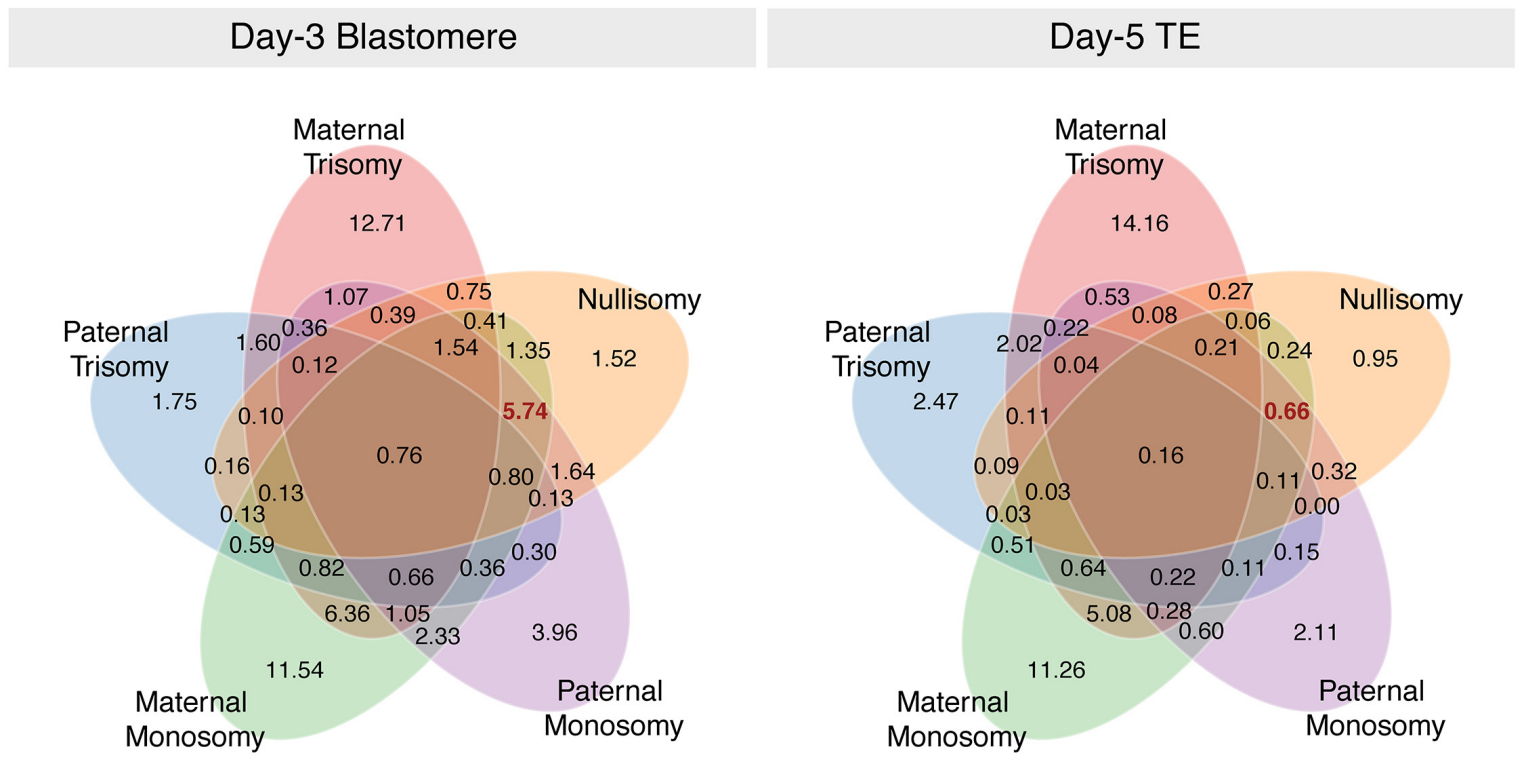
Venn diagram demonstrating that multiple forms of aneuploidy commonly co-occur within individual day-3 blastomeres. Numbers represent percent of the total sample. Complex aneuploidies, especially those involving multiple forms of chromosome loss (highlighted in red) are common at day 3, but rare at day 5 of development. Isolated errors, in contrast, are relatively viable through blastocyst formation.

Complex aneuploidies were non-random in their composition, with co-occurrence of certain forms of aneuploidy being more common than others (Fig 6). Maternal monosomy and maternal trisomy, the most prevalent forms of aneuploidy, frequently co-occurred within individual embryo biopsies at both day 3 and day 5. Aneuploidies involving multiple forms of chromosome loss (maternal monosomy, paternal monosomy, and nullisomy) were extremely prevalent at day 3 of development, but rare among day-5 biopsies, again suggesting strong selection against this common class of whole-chromosome abnormality (Fig 6).

### Characteristics of meiotic error

The diverse forms and distinct characteristics of whole-chromosome abnormalities revealed by these analyses motivated us to separately investigate errors of meiotic and mitotic origin to shed light on the cytogenetic mechanisms that produce these patterns and the consequences for reproduction and early development. Focusing first on meiotic errors, we analyzed rates of maternal and paternal BPH errors, their associations with parents’ ages, and their tendencies toward particular chromosomes.

**Fig 7:**
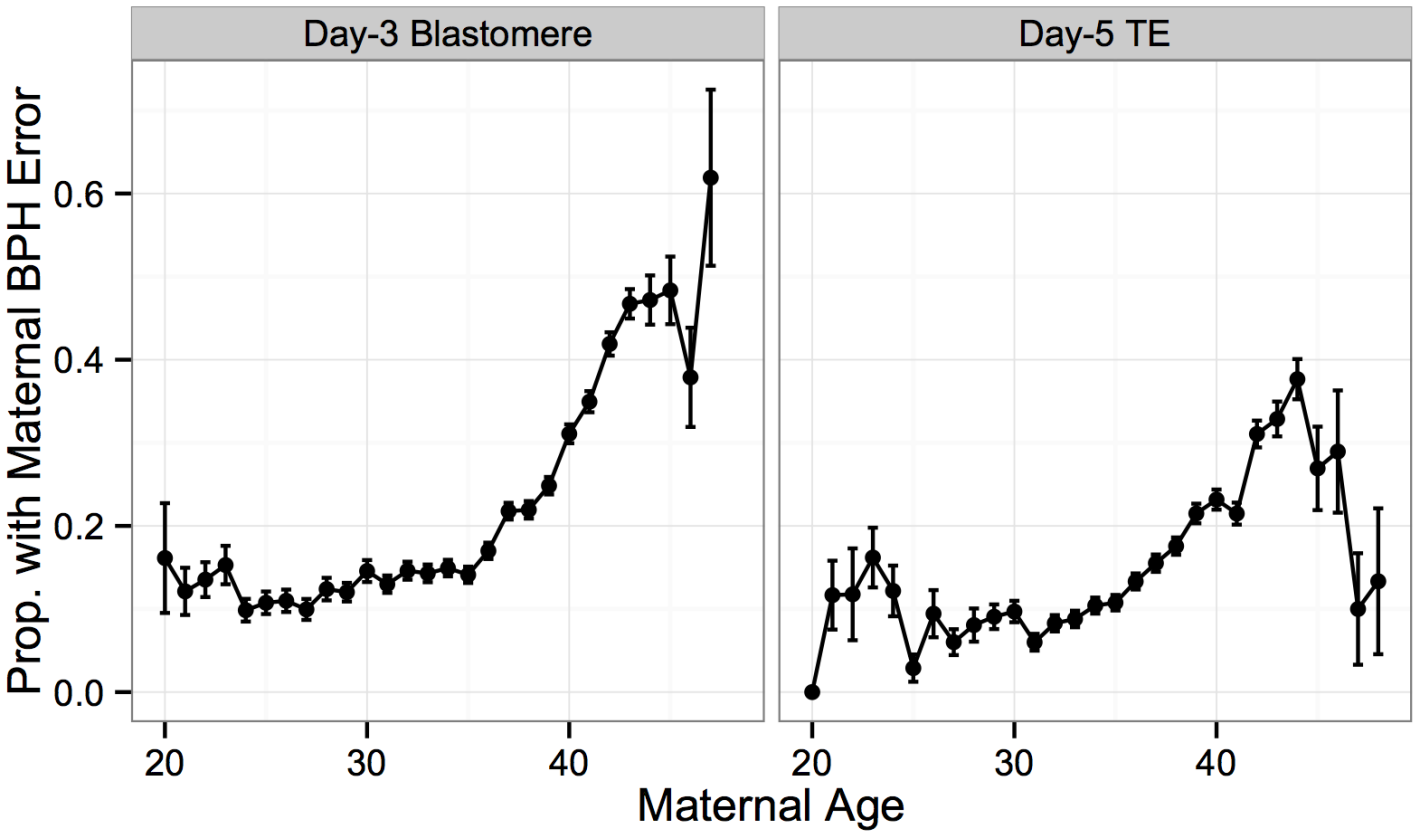
Maternal BPH error increases with maternal age. Proportion of biopsies containing at least one maternal BPH error, stratified by sample type. Maternal BPH error increases with maternal age for both day-3 blastomere biopsies (*β* = 0.110, *SE* = 0.00404, *P* < 1 × 10^−10^) and day-5 TE biopsies (*β* = 0.120, *SE* = 0.00599, *P* < 1 × 10^−10^). Error bars indicate standard errors of proportions.

Maternal BPH errors increased dramatically with maternal age, consistent with the interpretation that maternal meiotic errors drive the maternal age association with aneuploidy (Fig 7). This association was observed for both day-3 blastomere biopsies (*β* = 0.110, *SE* = 0.00404, *P* < 1 × 10^−10^) and day-5 TE biopsies (*β* = 0.120, *SE* = 0.00599, *P* < 1 × 10^−10^). Decreased maternal BPH error was also responsible for the surprisingly low rate of whole-chromosome abnormalities in TE biopsies from patients > 45 years old, described in the previous section (Figs 7 and 3).

**Fig 8:**
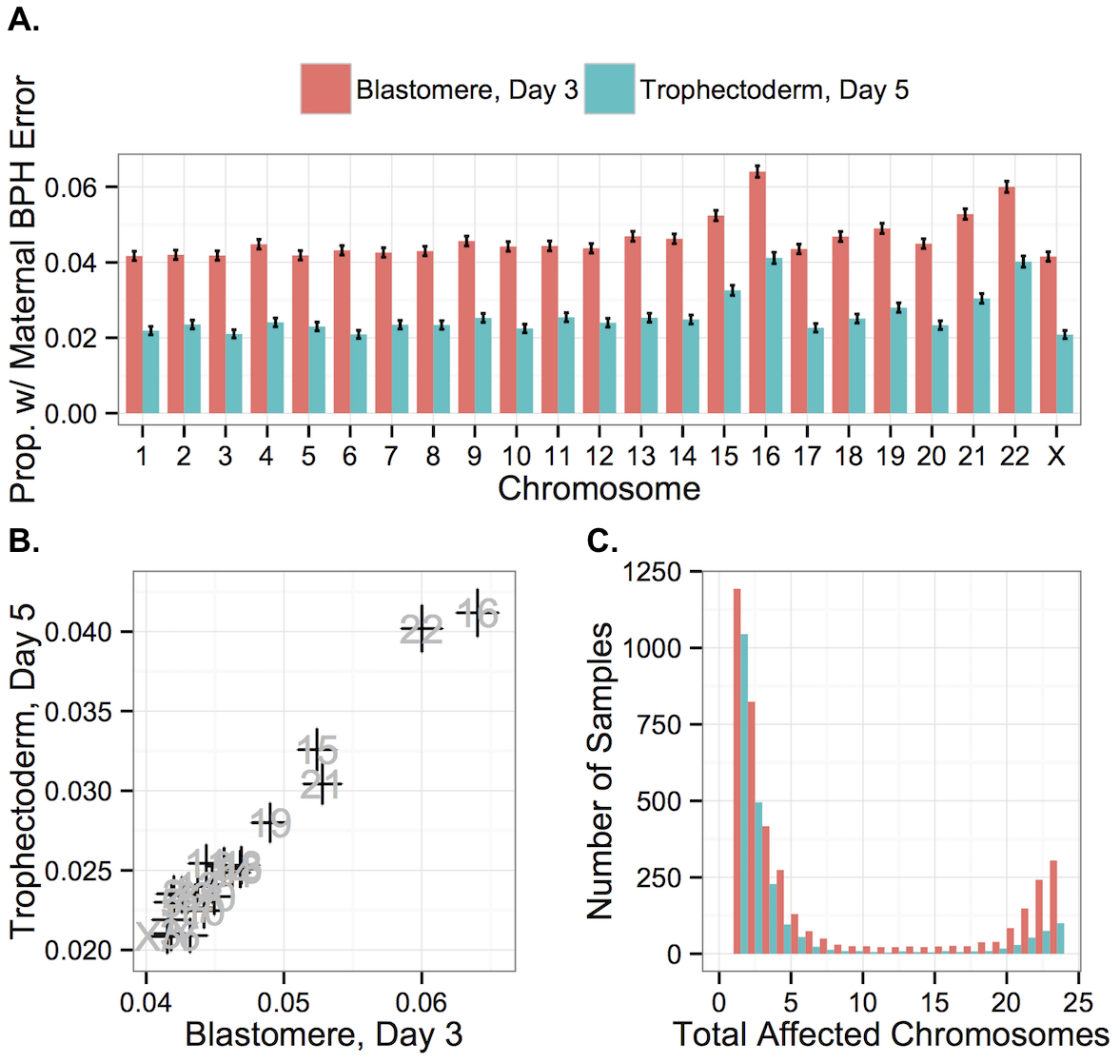
Rates of maternal BPH error are elevated on specific chromosomes. **A**: Chromosomes 16, 22, 21, and 15 displayed elevated rates of maternal BPH error at both day 3 and day 5. **B**: Chromosome-specific rates of maternal BPH error are highly correlated at day 3 versus day 5 (*r* = 0.978, *P* < 1 × 10^−10^). Error bars indicate standard errors of proportions. **C**: Histogram of total affected chromosomes for biopsies with at least one maternal BPH error, but no putative mitotic errors. Colors indicate sample type.

Maternal BPH errors did not affect all chromosomes equally, with elevated rates of BPH error of chromosome 16 (d3: 6.40%, d5: 4.12%), 22 (d3: 6.00%, d5: 4.02%), 21 (d3: 5.28%, d5: 3.04%) and 15 (d3: 5.24%, d5: 3.26%) (Fig 8A). A high rate of aneuploidy of these chromosomes is consistent with previous studies applying PGS to diverse developmental stages, from oocyte polar bodies [9, 43, 44] (though see [45] for why this might not reflect the status of the embryo) to products of conception from clinical miscarriages [46–51]. Together, these results support the suggestion these chromosomes are both inherently more susceptible to meiotic error and that meiotic errors are relatively viable into late development.

Chromosome-specific rates of maternal BPH trisomy and maternal monosomy were also highly correlated with one another (*r* = 0.840, *P* = 5.471 × 10^−7^), reflecting the fact that many maternal BPH trisomies and maternal monosomies likely share a common origin of unbalanced chromatid predivision. Further supporting this conclusion, chromosomes 16, 22, 15, and 21, which had the highest rates of maternal BPH trisomy and monosomy, also displayed the strongest increases with maternal age. This increase greatly exceeded the maternal age effects on other chromosomes (S3 Fig), corroborating recent findings by Franasiak et al. [25]. A generalized linear model confirmed the presence of a length-by-age interaction effect on probability of maternal BPH trisomy of particular chromosomes (*β* = 2.494 × 10^−10^, *SE* = 6.423 × 10^−11^, *P* = 1.15 × 10^−4^; S2 Table).

**Fig 9:**
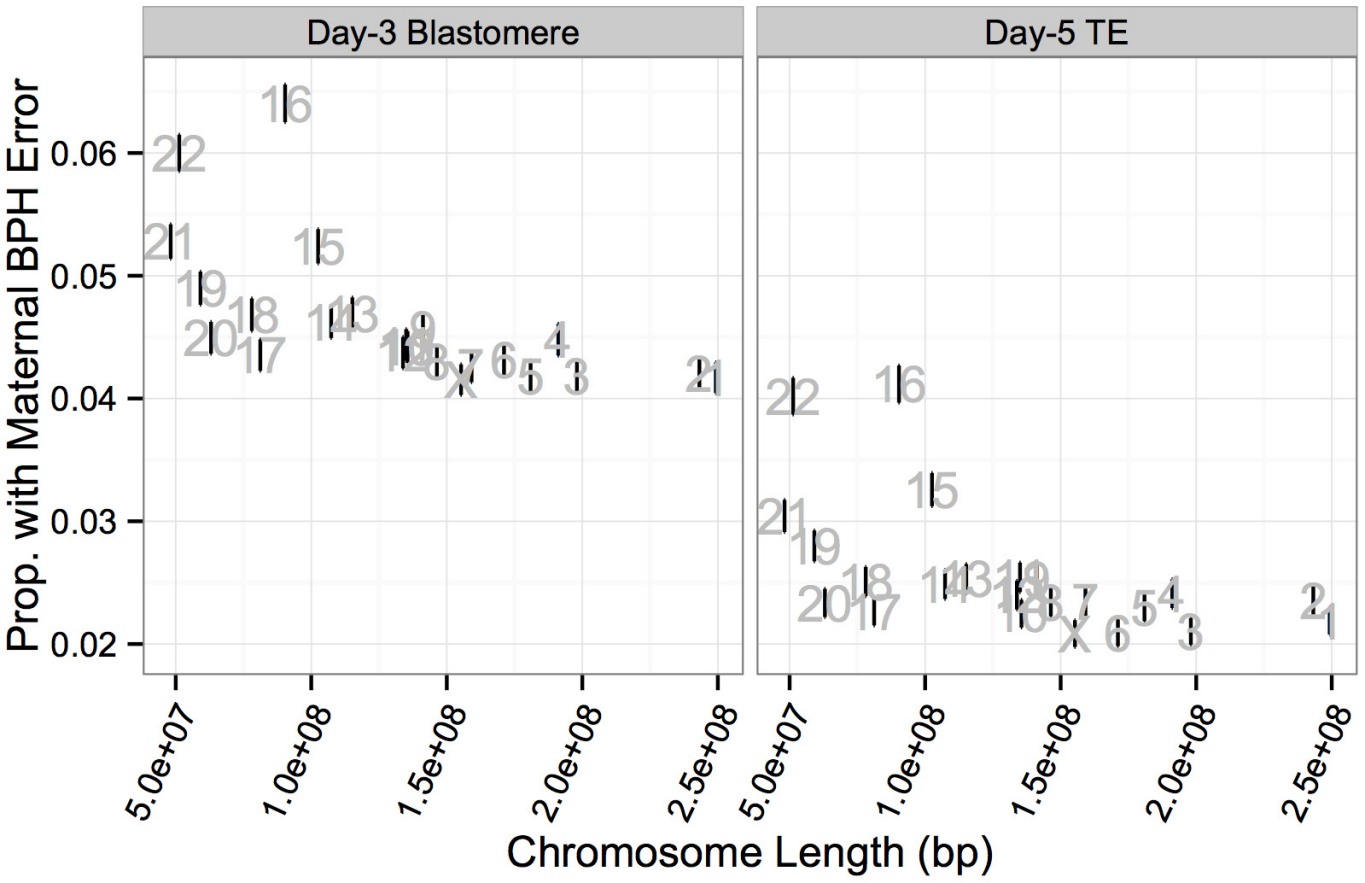
Chromosome-specific rates of maternal BPH aneuploidy are negatively correlated with chromosome length. Negative correlation was observed for both day-3 blastomeres (*r* = −0.623, *P* = 0.00148) and day-5 TE biopsies (*r* = −0.556, *P* = 0.00586). Error bars indicate standard errors of proportions.

While chromosome-specific rates of BPH error were lower at day 5 of development, this was almost exclusively due to selection against triploidies and haploidies (as well as near-triploidies and near-haploidies) rather than BPH aneuploidies affecting small numbers of chromosomes. Chromosome-specific rates of maternal BPH error at day 3 and day 5 were highly correlated (Fig 8B; *r* = 0.978, *P* < 1 × 10^−10^), consistent with weak selection against minor meiotic-origin aneuploidies between these stages. Chromosome-specific rates of maternal BPH error were also negatively correlated with chromosome length at both day 3 (Fig 9; *r* = −0.623, *P* = 0.00148) and day 5 (Fig 9; *r* = −0.556, *P* = 0.00586). This correlation was still significant after removing chromosomes 15, 16, 21, and 22 from the analysis (day-3 blastomeres: *r* = −0.734, *P* = 0.000346; day-5 TE biopsies: *r* = −0.525, *P* = 0.0209). Upon excluding co-occurring cases of putative mitotic error, we observed that maternal BPH errors very rarely affected intermediate numbers of chromosomes, instead tending toward few chromosomes (aneuploidy) or the entire complement (polyploidy; Fig 8C).

**Fig 10:**
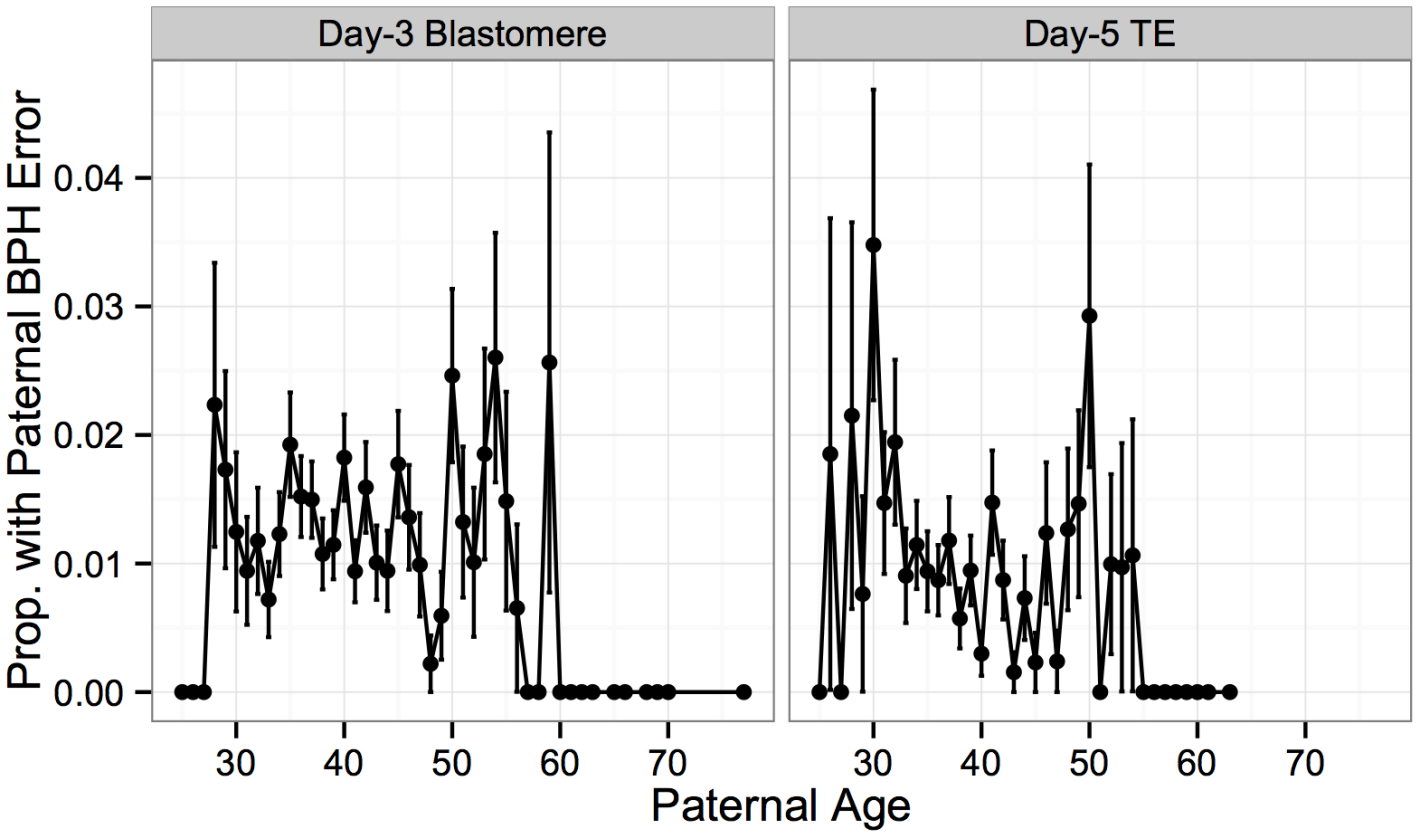
Association between paternal BPH error and paternal age. Rate of rare paternal BPH error was not associated with paternal age in day-3 blastomere biopsies (*β* = −0.00360, *SE* = 0.00933, *P* = 0.700), but displayed a weak negative association in day-5 TE biopsies (*β* = 0.0342, *SE* = 0.0146, *P* = 0.0194). Error bars indicate standard errors of proportions.

In contrast to frequent meiotic errors in the egg, meiotic errors in sperm are rare, with paternal BPH error detected in only 1% of biopsies in our study. No significant association was detected between paternal age and these rare paternal BPH errors in blastomere samples (Fig 10; Logistic GLM, *β* = −0.00360, *SE* = 0.00933, *P* = 0.700), while a significant, but weak, negative association was observed in TE biopsies (Fig 10; Logistic GLM, *β* = 0.0342, *SE* = 0.0146, *P* = 0.0194). These results suggest that residual correlation between maternal and paternal age, rather than increased susceptibility to paternal meiotic error, may have been responsible for the marginal positive association between aneuploidy and paternal age detected in our previous analysis.

### Characteristics of mitotic error

While the BPH signature can be used to identify chromosome gains of likely meiotic origin, mitotic errors must be identified by separate chromosomal signatures. Gain or loss of at least one paternal chromosome copy is a good indicator of mitotic error, as previous studies have determined that fewer than 5% of sperm are aneuploid [52] and paternal BPH error affected only 1% of samples in our study. This logic was recently used to classify mitotic errors in the same dataset, correlating their occurrence with a maternal effect genetic variant in the region containing the gene *PLK4* [37].

**Fig 11:**
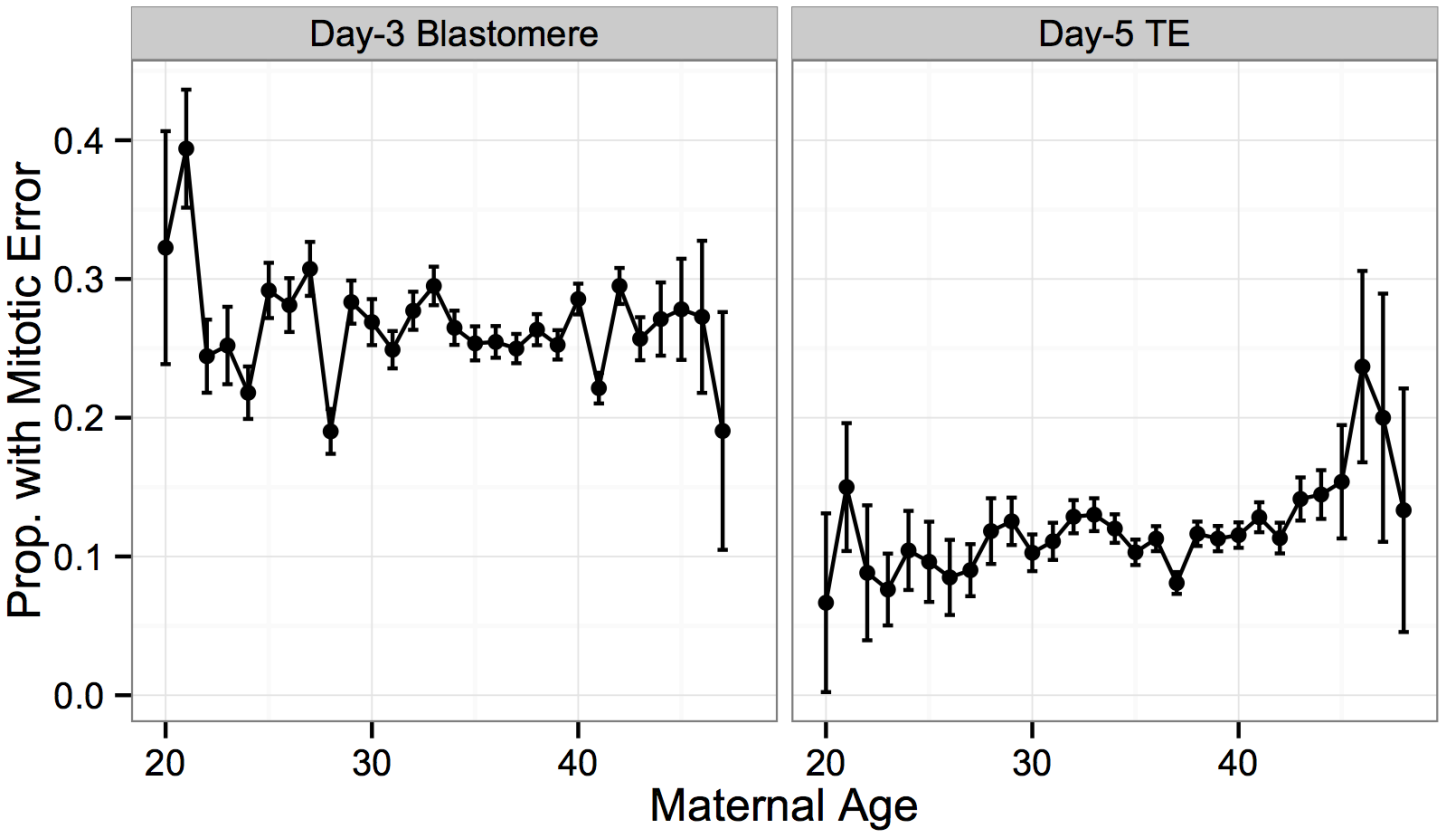
No significant association between putative mitotic error and maternal age. No significant association was detected between rate of putative mitotic error and maternal age for either day-3 blastomere biopsies (*β* = −0.00186, *SE* = 0.00322, *P* = 0.564) or day-5 TE biopsies (*β* = 0.0119, *SE* = 0.00616, *P* = 0.0526). Error bars indicate standard errors of proportions.

In contrast to extensive data supporting a maternal age effect on maternal meiotic-origin aneuploidy, previous studies have reached conflicting conclusions about whether mitotic error is influenced by maternal or paternal age. We observed that the incidence of mitotic error was not associated with maternal age for either day-3 blastomere biopsies (Fig 11; *β* = −0.00186, *SE* = 0.00322, *P* = 0.564) or day-5 TE biopsies (Fig 11; *β* = 0.0119, *SE* = 0.00616, *P* = 0.0526). This finding thus contradicts several previous studies [53–55], but is consistent with several others [56, 57]. While not statistically significant, the day-5 biopsies show a trend in the positive direction, and a more comprehensive approach to classifying mitotic error may reveal a statistically significant association. Nevertheless, the sample size of our study is so large that any effect, should it exist, would be extremely weak in comparison to the strong maternal age effect on meiotic error.

**Fig 12:**
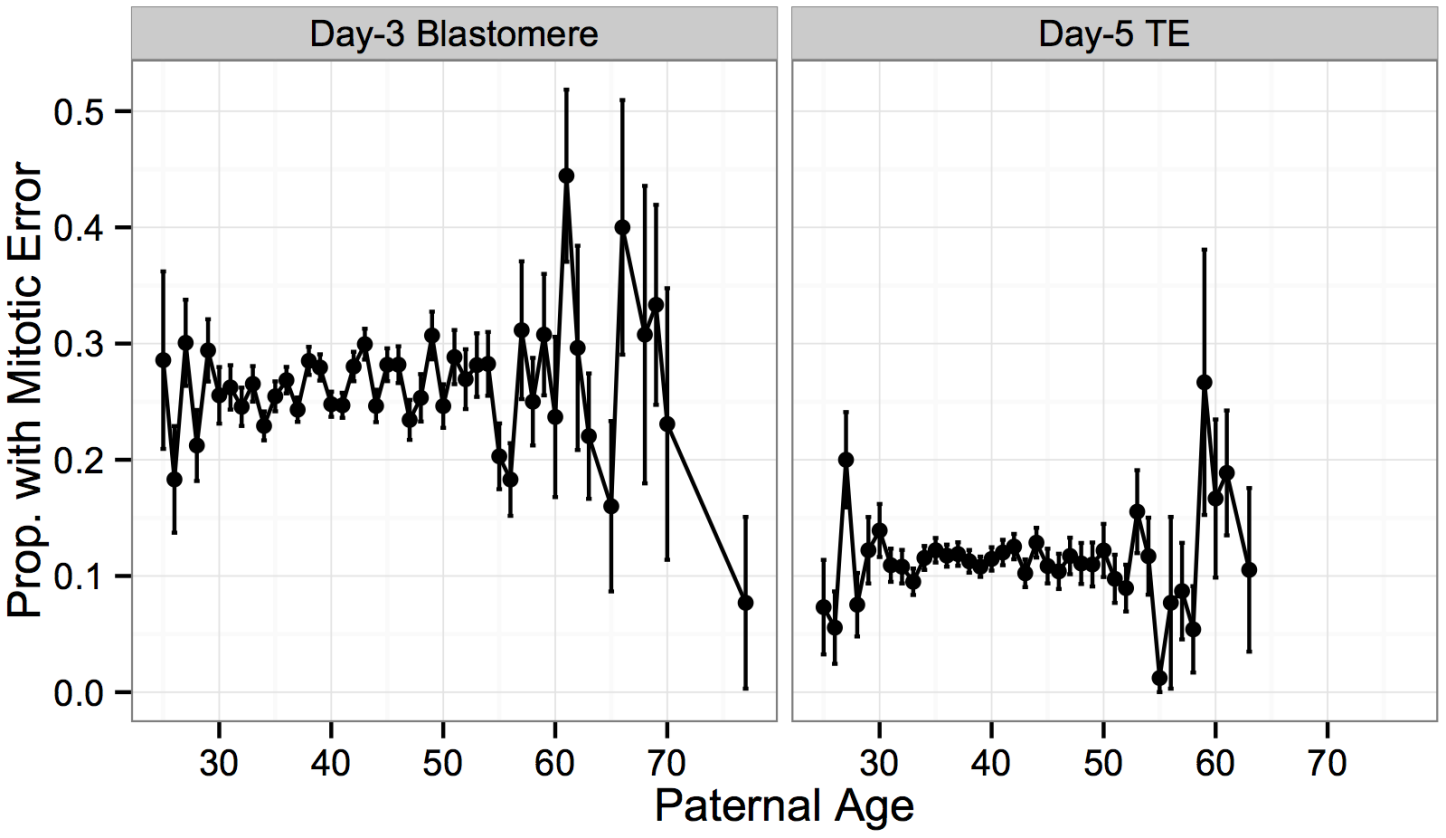
No significant association between putative mitotic errors and paternal age. No significant association was detected between rate of putative mitotic error and paternal age for either day-3 blastomere biopsies (*β* = 0.00315, *SE* = 0.00253, *P* = 0.213) or day-5 TE biopsies (*β* = 0.000540, *SE* = 0.00454, *P* = 0.905). Error bars indicate standard errors of proportions.

Similarly, no significant association was detected between incidence of mitotic error and paternal age at either day 3 (Fig 12; *β* = 0.00315, *SE* = 0.00253, *P* = 0.213) or day 5 (Fig 12; *β* = 0.000540, *SE* = 0.00454, *P* = 0.905). This finding again suggests that the weak paternal age effect on the rate of whole-chromosome abnormalities detected in our previous analysis, may indeed be driven by confounding effects of maternal age on maternal meiotic error.

**Fig 13:**
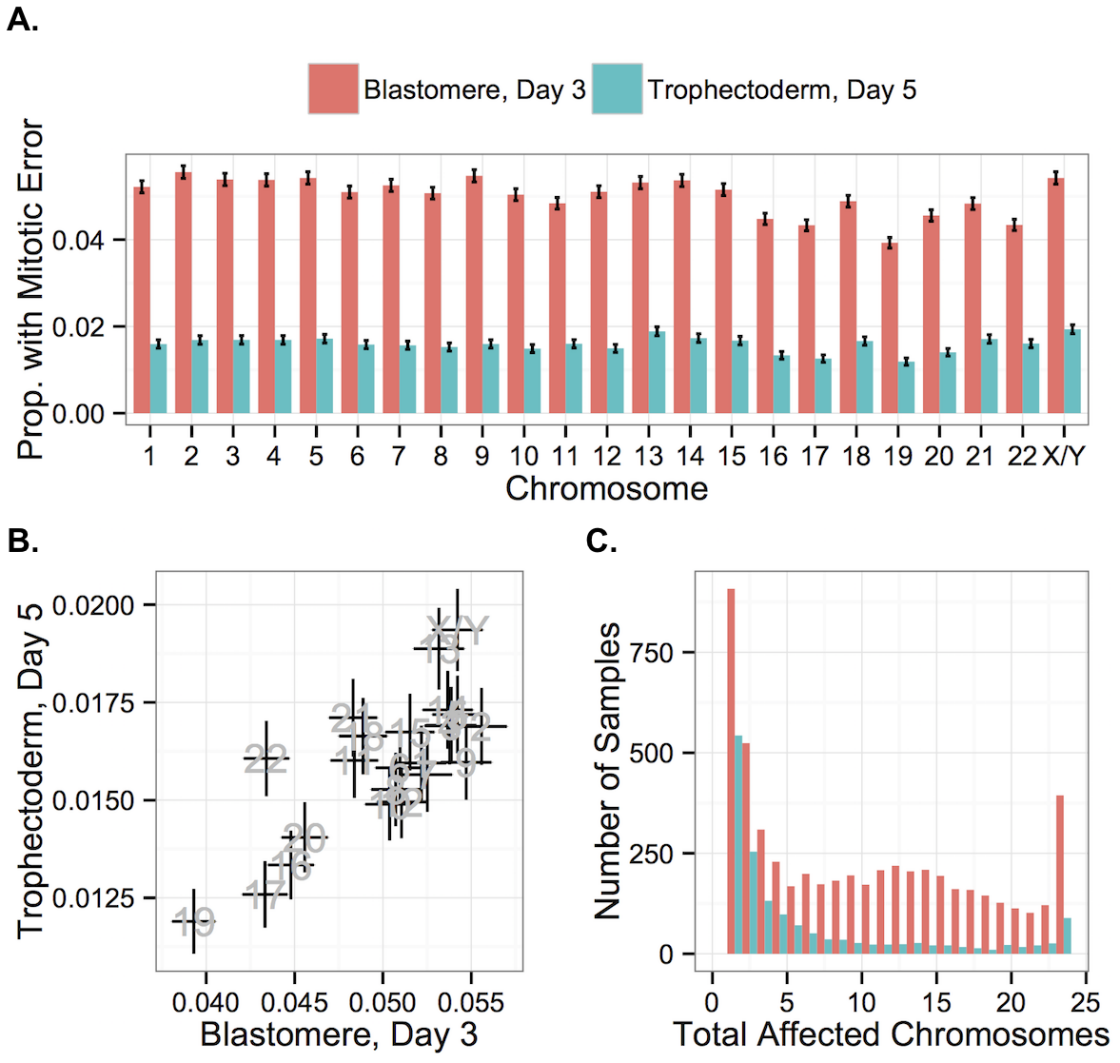
Chromosome-specific rates of aneuploidies of putative mitotic origin. **A**: Chromosome-specific rates of putative mitotic-origin aneuploidy vary by chromosome. **B**: Rates of putative mitotic-origin aneuploidy were significantly correlated between days 3 and 5 (*r* = 0.762, *P* = 2.326 × 10^−5^). Error bars indicate standard errors of proportions. **C**: Histogram of total aneuploid chromosomes for biopsies affected with putative mitotic-origin aneuploidy, but no maternal BPH aneuploidies. Colors indicate sample type.

**Fig 14:**
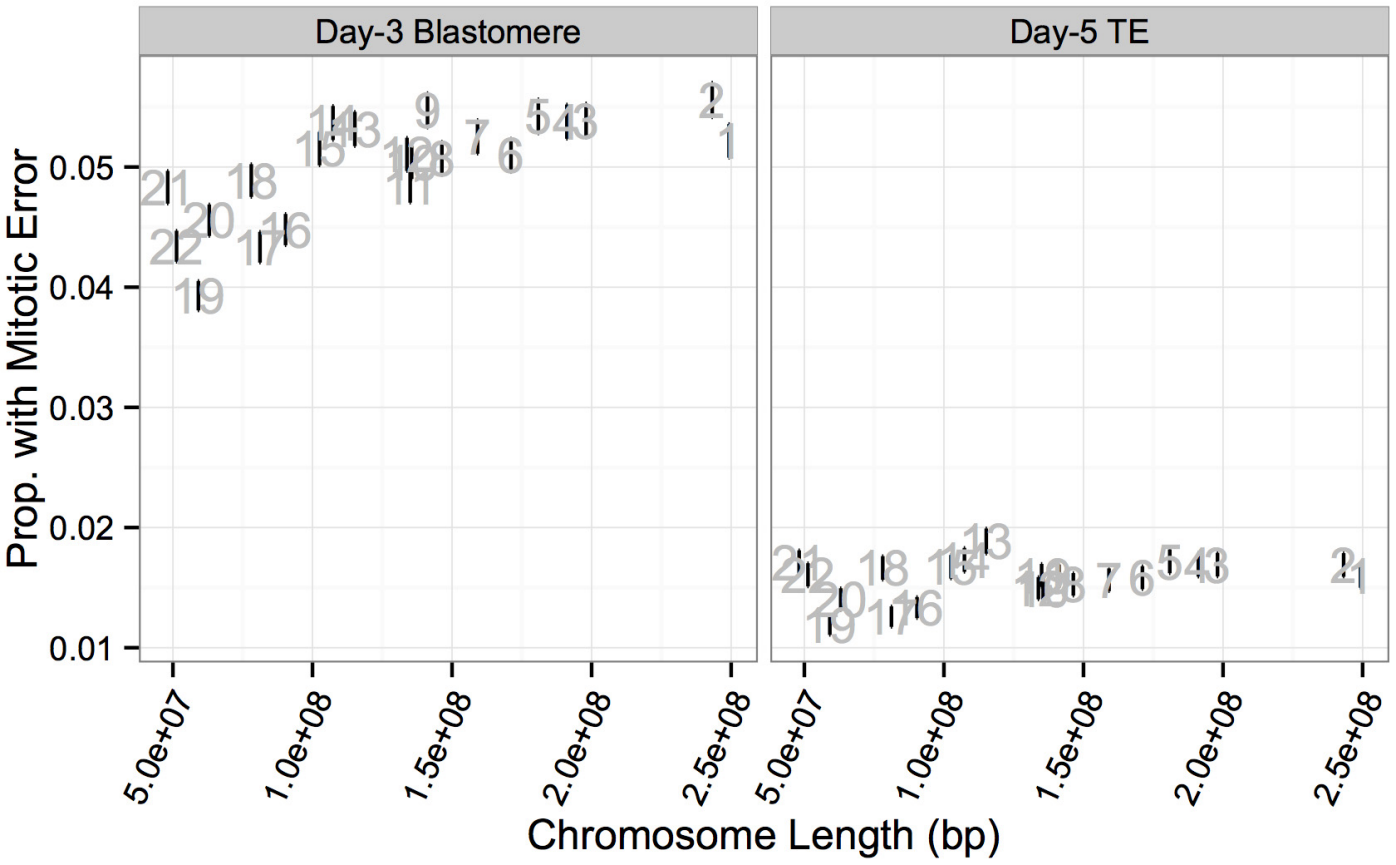
Chromosome-specific rates of putative mitotic errors are positively correlated with chromosome length in day-3 blastomeres. Positive correlation was observed for both day-3 blastomeres (*r* = 0.734, *P* = 1.011 × 10^−4^) but not in day-5 TE biopsies (*r* = 0.351, *P* = 0.109). Error bars indicate standard errors of proportions.

In contrast to meiotic errors, larger chromosomes were more susceptible to mitotic error, with increased mitotic error rates observed for these chromosomes at day 3 (Figs 13A and 14; *r* = 0.734, *P* = 1.011 × 10^−4^), but not at day 5 (Figs 13A and 14; *r* = 0.351, *P* = 0.109). Despite a strong depletion of mitotic-origin aneuploidies between days 3 and 5, chromosome-specific rates of mitotic error were still positively correlated between these developmental stages (Fig 13B; *r* = 0.762, *P* = 2.326 × 10^−5^). Unlike meiotic errors, which tended to affect few chromosomes or the entire complement, mitotic errors frequently affected intermediate numbers of chromosomes in blastomere biopsies, but not in TE biopsies (Fig 13C).

Current evidence suggests that post-zygotic mitotic errors primarily arise via mechanisms termed anaphase lag, mitotic non-disjunction, and endoreplication [56, 58–61], but the relative frequencies of these mechanisms are the subject of debate [13]. Endoreplication refers to genome duplication without cell division, resulting in binucleate blastomeres and balanced polyploidy that is undetectable with our SNP microarray approach (and would also be indistinguishable from normal mitotic cells immediately following S-phase) [61]. Anaphase lag occurs due to failure of chromatids to connect to the mitotic spindle or due to slow migration of chromatids toward the spindle poles, resulting in chromosome loss or incorporation into micronuclei [62]. Mitotic non-disjunction is similar to meiotic non-disjunction and refers to the failure of sister chromatids to separate during mitotic anaphase, resulting in a trisomy in one daughter cell with a corresponding monosomy in the other daughter cell.

The counts of mitotic chromosome gains and losses can thus be compared to assess relative frequencies of mitotic error mechanisms. Previous studies have argued that a predominance of chromosome loss compared to chromosome gain can be attributed to a high rate of anaphase lag [60, 63]. On a per-chromosome basis, we observed that chromosome losses indeed exceeded chromosome gains 54,626 to 12,907 for samples affected by putative mitotic errors. Our data, however, suggest that these chromosome losses are not primarily due to isolated cases of anaphase lag, but tend to be driven by more catastrophic errors in mitosis. These aneuploidies may be driven by centrosome or mitotic spindle abnormalities [64] and are consistent with chaotic cell division reported in previous studies [59]. The discovery that variation encompassing the gene *PLK4* influences mitotic-origin aneuploidy provides one clue that dysregulation of centrosome duplication may be an important factor underlying spindle abnormalities and aneuploidy in cleavage-stage embryos [37]. Centrosome overduplication, as is induced by overexpression of *PLK4*, can result in multipolar cell division [65] or centrosome clustering and a high rate of anaphase lag [66], with both mechanisms having the potential to cause multiple chromosome loss.

### Selection against complex aneuploidy

**Fig 15:**
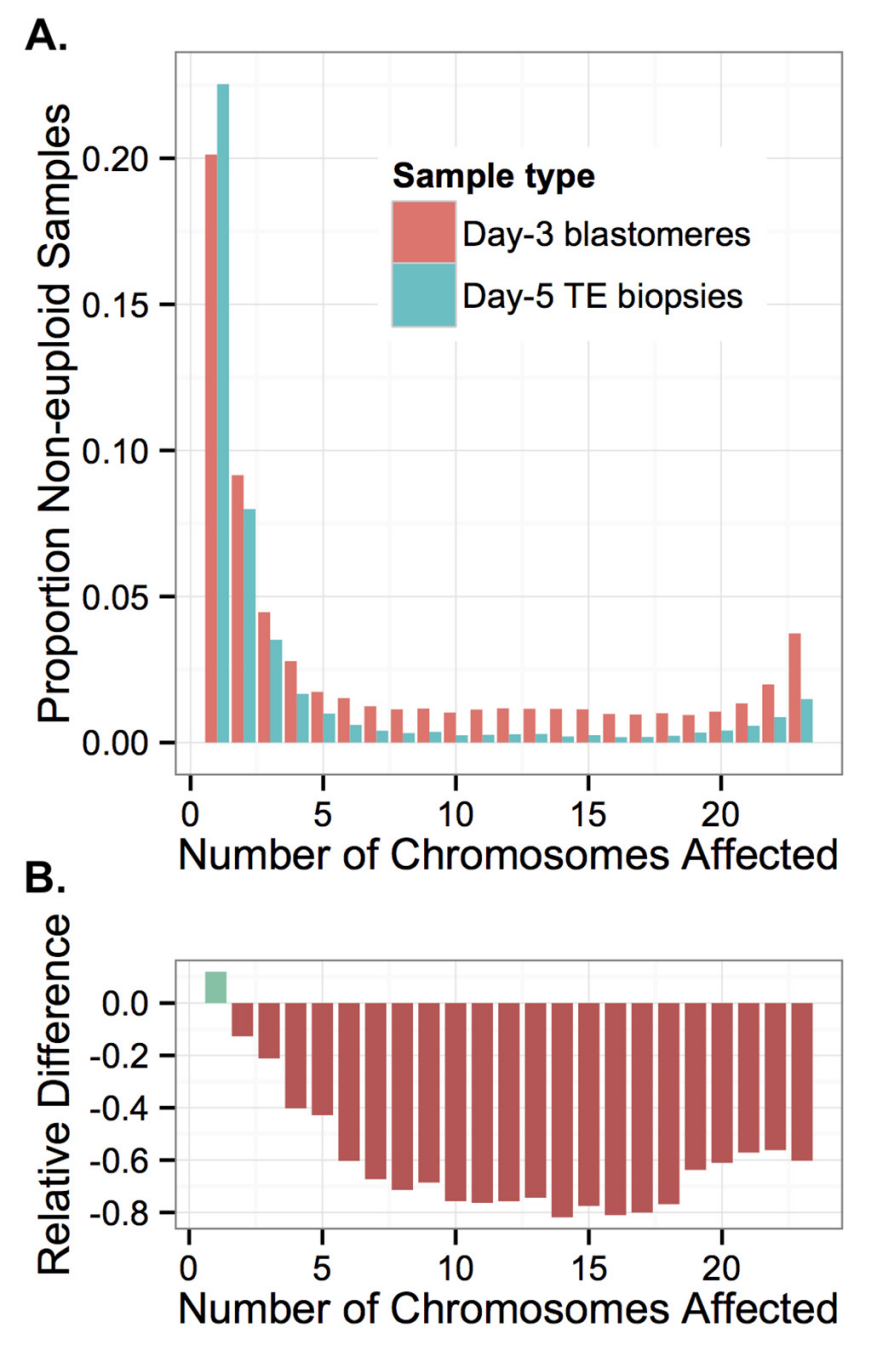
Complex errors are more common in blastomere samples than TE samples. **A**: Rate of non-euploidy according to total number of chromosomes affected, stratified by sample type. **B**: The relative difference between rates of non-euploidy affecting TE versus blastomere samples. More complex errors affecting greater numbers of chromosomes are increasingly rare among TE samples, suggesting inviability and/or self-correction of increasingly complex errors.

We next explicitly contrasted the two developmental stages to gain additional insight into selection occurring during preimplantation development. While a plurality of errors affected only one chromosome, greater than 80% of errors in day-3 blastomeres affected two or more chromosomes (Fig 15A). Compared to individual day-3 blastomere samples, fewer complex errors affecting multiple chromosomes were detected in day-5 TE biopsies (Fig 15A; Table 2).

We compared the two sample types by calculating the percent difference in rates of non-euploidy between the blastomere and TE samples, stratifying by the total number of affected chromosomes (Fig 15B). This metric reflects the proportion of embryos that were either lost or self-corrected between the two sampling stages. Due to the design of our study, we could not distinguish between embryonic arrest and self-correction, as blastomere and TE biopsy data were fully independent. Nevertheless, we observed that errors affecting increasing numbers of chromosomes were increasingly depleted in TE biopsies relative to blastomeres, plateauing at approximately 11 chromosomes affected (Fig 15B). This difference became less extreme when greater than 18 chromosomes were affected (Fig 15B), suggesting a slight relative viability of polyploidies compared to complex aneuploidies. Together, these results provide strong evidence of early selection against complex aneuploidy of primarily mitotic-origin.

### Association between whole-chromosome abnormalities and clinical indications

**Fig 16:**
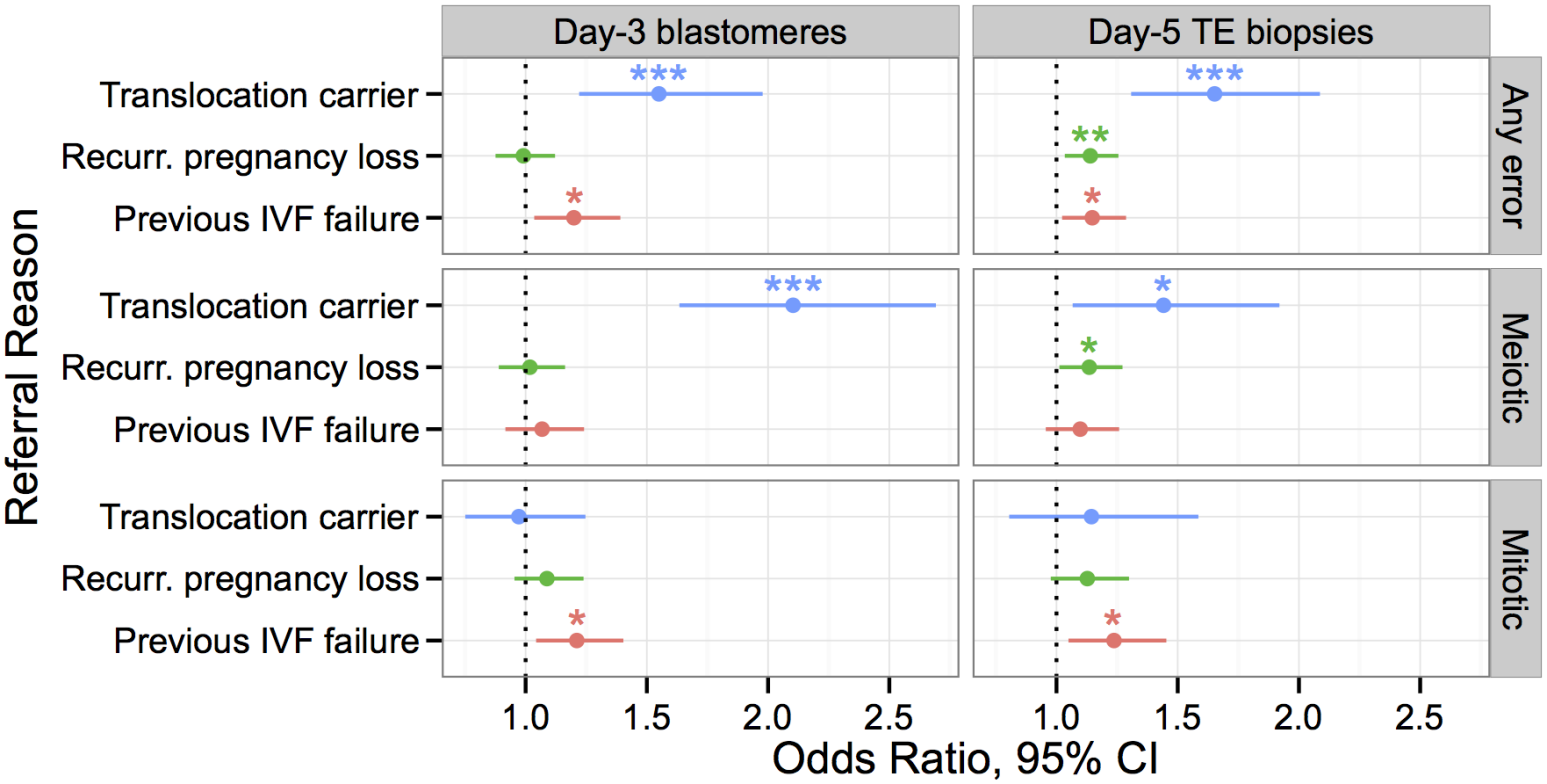
Associations between clinical indications for PGS and rates of meiotic and mitotic error detected with PGS, controlling for maternal age. Only indications with at least one significant association are depicted, while full results are provided in S3–S8 tables. Effect size is measured by an odds ratio, where error incidence for a given referral reason is compared to error incidence for all other referral reasons. Error bars indicating 95% confidence intervals. Stars are used to indicate statistical significance in a logistic GLM: * *P* < 0.05, ** *P* < 0.01, *** *P* < 0.001. Translocation carriers had significantly higher rates of meiotic error than patients referred for other reasons. Patients with previous IVF failure had higher rates of mitotic, but not meiotic error, while patients with recurrent pregnancy loss had higher rates of meiotic (BPH) error at day 5.

Our analysis revealed distinct characteristics of whole-chromosome abnormalities generated by meiotic versus mitotic mechanisms. Variation in meiotic and mitotic error rates in embryos from different parents is likely due to a wide array of environmental and genetic factors, not least of which are age [3] and *PLK4* genotype [37]. Several researchers have noted a striking elevation of aneuploidy rates in embryos from particular patients, unrelated to maternal age [59]. Together, these findings led us to hypothesize that different forms of infertility—and thus, different referral reasons—would be specifically associated with increased rates of either meiotic or mitotic error. We therefore tested observed rates of meiotic and mitotic error against reasons for PGS referral, regressing out the effect of maternal age where appropriate (Fig 16).

Chromosomes of parental carriers of reciprocal translocations form quadrivalent structures during meiosis [67, 68]. A fraction of their gametes will thus be unbalanced and the resulting embryos are generally inviable due to these meiotic errors. Known carriers of translocations had expectedly higher rates of whole-chromosome abnormalities in both day-3 blastomere biopsies (*β* = 0.438, *SE* = 0.123, *P* = 0.00038) and day-5 TE biopsies (*β* = 0.502, *SE* = 0.119, *P* = 2.57 × 10^−5^) when compared to other clinical indications. Consistent with the known etiology of aneuploidy susceptibility in these patients, translocation status was associated with increased rates of meiotic (day-3 blastomeres: *β* = 0.743, *SE* = 0.127, *P* = 6.54 × 10^−9^; day-5 TE biopsies: *β* = 0.366, *SE* = 0.150, *P* = 0.0146), but not mitotic error (day-3 blastomeres: *β* = −0.0285, *SE* = 0.129, *P* = 0.826; day-5 TE biopsies: *β* = 0.135, *SE* = 0.172, *P* = 0.435).

Previous studies have also demonstrated an association between IVF failure and patient-specific rates of embryonic aneuploidy [69]. Our data replicate this result, as previous IVF failure was associated with increased error rates in both day-3 blastomere biopsies (*β* = 0.181, *SE* = 0.0752, *P* = 0.0160) and day-5 TE biopsies (*β* = 0.138, *SE* = 0.0585, *P* = 0.0187). Given our previous results suggesting selection against putative mitotic-origin aneuploidies between days 3 and 5, we hypothesized that this association would be driven by errors of mitotic origin. Consistent with this hypothesis, previous IVF failure was associated with increased rates of mitotic error in both day-3 (*β* = 0.191, *SE* = 0.0756, *P* = 0.0114) and day-5 embryos (*β* = 0.213, *SE* = 0.0830, *P* = 0.0104), but not associated with meiotic error (day-3 blastomeres: *β* = 0.0656, *SE* = 0.0773, *P* = 0.396; day-5 TE biopsies: *β* = 0.00934, *SE* = 0.00701, *P* = 0.183). Given the recent finding that *PLK4* influences rates of mitotic-origin aneuploidy, we also tested maternal *PLK4* genotype for association with previous IVF failure, but found no significant signal of this transitive association (*β* = 0.0719, *SE* = 0.0826, *P* = 0.384). The wide confidence interval around the coefficient estimate (95% CI [-0.091, 0.233]), however, reflects the small sample size and does not rule out the existence of a moderate effect, such as might be expected given the *PLK4* effect size and the relatively modest association between mitotic error and IVF failure.

While mitotic error likely contributes to preclinical pregnancy loss, nearly all whole-chromosome abnormalities observed in products of conception from clinical miscarriages are attributable to meiotic error [3]. We thus hypothesized that recurrent pregnancy loss would be associated with increased rates of meiotic error, even after controlling for the well-documented maternal age effect. Recurrent pregnancy loss was indeed associated with increased error rates in day-5 TE biopsies (*β* = 0.131, *SE* = 0.0494, *P* = 0.00831), but not in day-3 blastomeres (*β* = −0.00945, *SE* = 0.0630, *P* = 0.880), possibly due to limitations in sample size and the predominance of mitotic errors at this developmental stage. Consistent with our hypothesis, the day-5 TE association was driven by an underlying association with meiotic (*β* = 0.127, *SE* = 0.0584, *P* = 0.0299), but not mitotic error (*β* = 0.120, *SE* = 0.0728, *P* = 0.100).

## Discussion

Our study is the largest genetic survey of IVF embryos to date. By leveraging parent and embryo genotypes measured via SNP-microarray, we inferred ploidy of all 24 chromosomes of embryo biopsies and assigned detected errors to individual parental homologs. This allowed us to classify whole-chromosome abnormalities of putative meiotic and mitotic origin to separately investigate parental age associations and chromosome-specific profiles. Our results demonstrate that this classification approach is important, given the diversity of cytogenetic mechanisms, karyotypic profiles, and consequences for preimplantation development. Our data support the current understanding that whole-chromosome abnormalities are primarily due to errors in maternal meiosis, as well as frequent mitotic errors arising during post-zygotic cell division. Variation in rates of mitotic error may be explained in part by maternal factors (e.g. [37]), as the initial post-zygotic cell divisions are controlled by maternal gene products [70]. In comparison to maternal meiotic and mitotic errors, we found that paternal meiotic errors were rare, though they are of demonstrated importance in some cases [71]. This is consistent with the finding that relatively common male infertility (affecting approximately 7% of men) is primarily due to factors other than whole-chromosome abnormalities [72].

Errors did not affect all chromosomes equally. Maternal BPH (meiotic) trisomies and maternal monosomies were correlated in their bias toward smaller chromosomes, consistent with common mechanisms of formation. Both non-disjunction and unbalanced chromatid predivision produce corresponding chromosome gains and losses. While these mechanisms are indistinguishable in our data, unbalanced chromatid predivision has been shown to be much more common [9–11], and a study of metaphase II oocytes and corresponding polar bodies found that this mechanism tended to affect smaller chromosomes [73]. A similar study found that these predivision errors were most strongly affected by maternal age [74]. Thus, inherent susceptibility of smaller chromosomes to premature separation of sister chromatids is likely responsible for the chromosome-specific age association observed in our study. Recent results from a large survey of TE biopsies [75] also support this interpretation, as this study detected a disproportionate maternal age effect on aneuploidies of chromosomes 13, 15, 16, 18, 19, 21, and 22. Our study is the first, however, to characterize chromosome-specific rates of putative mitotic errors based on a large sample, demonstrating that these errors have a modest, but statistically significant bias toward larger chromosomes.

Our study replicated the well-documented association between maternal age and incidence of maternal meiotic error. Using complementary statistical approaches, we also detected a significant association between paternal age and day-3 blastomere aneuploidy while controlling for the correlated effect of maternal age. By stratifying errors of putative maternal meiotic, paternal meiotic, and mitotic origin, however, we demonstrated that the paternal age association is likely driven by maternal meiotic error. Given the small effect size and the lack of plausible biological mechanism for such an association, we conclude that the paternal age effect is likely a statistical artifact due to residual correlation between maternal and paternal ages. This may help explain conflicting results in previous literature [35]. We also detected a weak negative association between paternal age and paternal BPH error in day-5 TE biopsies, but note that a recent study of trisomy 21 also found a negative association [76].

The central finding of our study was a high incidence of complex mitotic-origin aneuploidies in day-3 blastomere biopsies, which we conclude are purged by selection preceding blastocyst formation. Complex mitotic-origin aneuploidies were strongly biased toward chromosome losses over chromosome gains, and tended to involve random combinations of maternal monosomy, paternal monosomy, and nullisomy. This error profile strongly suggests a post-zygotic mechanism that does not discriminate among maternal and paternal homologs. Blastomeres containing such aneuploidies may have been sampled from mosaic embryos undergoing chaotic cell division due to centrosome abnormalities or other mitotic aberrations [5]. Complex mitotic-origin aneuploidies are under-appreciated in the literature, potentially due to technical limitations of alternative PGS technologies. FISH, for example, could systematically underestimate the extent of these errors because ploidy statuses of only a few chromosomes are assayed at once. Chromosome losses are also difficult to distinguish from FISH hybridization failure, potentially causing many complex mitotic-origin aneuploidies involving multiple chromosome losses to be falsely attributed to technical artifacts. Similarly, aCGH cannot distinguish between maternal or paternal identity of homologs and lacks resolution to distinguish meiotic and mitotic errors from single biopsies.

One important limitation of our study is the fact that mitotic-origin aneuploidies present in diploid-aneuploid mosaic embryos may not be detectable if impacting a small proportion of cells in a biopsy. As intra-sample mosaicism is only present in multi-cell day-5 TE biopsies, this could confound our interpretation that selection purges these aneuploidies prior to blastocyst formation. While this limitation may be overcome by novel methods to detect mosaicism in multi-cell DNA extractions, we stand by our interpretation based on supporting results from previous disaggregation studies. Early applications of FISH-based PGS consistently demonstrated that extensive mosaicism was present at a higher rate in arrested embryos than in embryos surviving to the blastocyst stage [22, 26, 77–79]. We believe that these FISH results are trustworthy despite the technical limitations of the technology [80], as these limitations should not systematically affect arrested versus non-arrested embryos. Because these studies were limited to assaying few chromosomes, however, they lacked resolution to characterize the full extent of mitotic errors. Consistent with our finding that mitotic errors often affect large numbers of chromosomes, recent results from a survey of 385 cleavage-stage embryos found that survival to blastocyst formation correlates with the number of chromosomal abnormalities [81]. A previous microarray-based study by Natera [82] showed perfect concordance in ploidy between disaggregated TE fractions, again supporting the conclusion that mosaicism is rare on a per-cell (but not per-embryo [18, 83]) basis by the blastocyst stage.

In line with these PGS results, a high rate of mitotic spindle and cell division abnormalities have been documented in early embryos by independent methods, but embryos with aberrant spindles and abnormal cell division rarely survive to blastocyst formation. One previous study used confocal laser scanning microscopy to show that mitotic spindle abnormalities and multiple chromosome loss affect a large proportion of cleavage-stage embryos [64]. The proportion of normal mitotic spindles detected in this study increased from 50% at the cleavage stage to 87% at the blastocyst stage [64], again consistent with our observation that mitotic-origin aneuploidies are common in day-3 blastomeres but rare in day-5 TE biopsies. A recent study used time-lapse imaging to show that 0 of 18 two-pronuclear (2PN) zygotes that underwent aberrant multipolar cell division survived to the blastocyst stage [65, 84].

Strong, efficient selection against complex mitotic error means that these errors will rarely, if ever, contribute to clinical miscarriage. This does not imply that these errors are unimportant. Indeed, leveraging patient referral data, we detected a significant association between previous IVF failure and incidence of putative mitotic error. This finding in turn suggests that post-zygotic mechanisms of aneuploidy formation are an important factor limiting human fertility that may also help explain fertility differences among individuals. The latter suggestion is supported by the recent identification of a maternal genetic variant influencing mitotic-origin aneuploidy risk [37].

Fewer than 30% of conceptions progress to live birth, even for young, otherwise fertile couples [1]. Our findings highlight one reason for this low rate of human fertility, providing evidence that complex mitotic-origin aneuploidies abound in cleavage-stage embryos, but are purged early in preimplantation development.

## Materials and Methods

Laboratory and bioinformatic steps to determine the ploidy status of each embryo biopsy were performed by Natera, while statistical analyses and interpretation were primarily conducted by co-authors at Stanford University with input from Natera co-authors.

### Cell isolation, DNA amplification, and genotyping

Embryos were vitrified at the IVF clinics, shipped to Natera on dry ice, and analyzed within two weeks of arrival. Genetic material was obtained from oocyte donors (buccal swabs), fathers (peripheral venipuncture), and embryos (either single-cell day-3 blastomere biopsy or multi-cell day-5 trophectoderm biopsy). Single tissue culture and egg donor buccal cells were isolated using a sterile tip attached to a pipette and stereomicroscope (Leica; Wetzlar, Germany). For fresh day-3 embryo biopsy, individual blastomeres were separated via micromanipulator after zona pellucida drilling with acid Tyrode’s solution. Single cells for analysis were washed four times with buffer (PBS buffer, pH 7.2 (Life Technologies, 1mg/mL BSA). Multiple displacement amplification (MDA) with proteinase K buffer (PKB) was used for this procedure. Cells were placed in 5*μ*l PKB (Arcturus PicoPure Lysis Buffer, 100 mM DTT, 187.5 mM KCl, 3.75 mM MgCl_2_, 3.75 mM Tris-HCl) incubated at 56°C for 1 hour, followed by heat inactivation at 95°C for 10 min, and held at 25°C for 15 min. MDA reactions were incubated at 30°C for 2.5 hours and then 65°C for 10 min. Genomic DNA from buccal tissue was isolated using the QuickExtract DNA Extract Solution (Epicentre; Madison, WI). Template controls were included for the amplification method. Amplified single cells and bulk parental tissue were genotyped using the Infinium II (Illumina; San Diego, CA) genome-wide SNP arrays (HumanCytoSNP12 chip). The standard Infinium II protocol was used for parent samples (bulk tissue), and Genome Studio was used for allele calling. For single cells, genotyping was accomplished using an Infinium II genotyping protocol.

### Ploidy inference

Detection and classification of various forms of whole chromosome abnormality was achieved using the Parental Support algorithm previously described by Johnson et al. [6]. This approach uses high-quality genotype data from the father and the mother (or oocyte donor) to infer the presence or absence of homologs in embryo genotype data. This procedure is useful because embryo biopsies incur a high allelic dropout rate due to limited starting material and whole-genome amplification. Johnson et al. [6] performed validation of the Parental Support method on single cells biopsied from both aneuploid and euploid cell lines, comparing the approach to the ‘gold standard’ of metaphase karyotyping. The authors found that the sensitivity (97.9%; 323/330) and specificity (96.1%; 125/129) of the SNP-microarray based Parental Support approach were comparable to metaphase karyotyping. Furthermore, confidence scores obtained from this approach were strongly correlated with false-detection rates. Consistent with these results, Treff et al. [80] demonstrated that SNP-microarray-based approaches are more consistent in detecting aneuploidy than widely-used FISH technology.

Ploidy detection in day-5 blastocysts is complicated by the possibility of mosaicism within a single TE biopsy. Nevertheless, several studies have suggested that while the overall rate of mosaicism in blastocysts is higher than in individual blastomeres, due to the greater number of cells, the proportion of cells affected is much lower. We consider this limitation more extensively in the Discussion section.

### Statistical analyses

All statistical analyses were conducted using the R statistical computing environment [85]. Separate analyses were performed on day-3 blastomere biopsies and day-5 TE biopsies, which had different proportions of specific forms of whole-chromosome abnormalities.

We used Poisson regression to test for association between the numbers of samples submitted per case and maternal age. In order to model overdispersion, we did not fix the dispersion parameter (i.e. quasi-Poisson). We used logistic regression to test for associations between maternal and paternal ages and specific forms of meiotic and mitotic error. For each IVF case, we counted the number of embryos in which a particular form of whole-chromosome abnormality was detected, while considering all other embryos as controls. For the model of overall rate of whole-chromosome abnormalities, we added polynomial terms for maternal age in increasing order until the addition of a higher order term did not provide significantly better fit, as indicated by an F-test. In order to model overdispersion, we did not fix the dispersion parameter in these generalized linear models (GLMs) (i.e., quasi-binomial). We added egg donor status as a predictor to the best-fit age models, comparing the respective models with F-tests.

To test various clinical indications against rates of whole-chromosome abnormalities, we utilized complementary procedures to ensure that our findings were robust. We first fit a model of maternal age versus error rates, including polynomial terms for maternal age in increasing order until the addition of a higher order term did not provide significantly better fit. We then added to this model each clinical indication, testing whether addition of that clinical indication provided significantly better fit using an F-test. We also performed backward model selection with the Akaike information criterion (AIC), starting with a full model that included all clinical indications (except for advanced maternal age, as maternal age was separately included in the model). Fig 16 depicts the results from the full regression model, as all significant predictors (*P* < 0.05) were also significantly associated when tested using the complementary statistical procedures described above. Regression coefficients (*β*) were exponentiated to calculate odds ratios.

We used Pearson correlations to assess the relationships between chromosome-specific rates of error affecting different developmental stages and chromosomes with different lengths as well as the relationship between chromosome-specific rates of different forms of whole-chromosome abnormality.

To test for an interaction between chromosome-specific rates of maternal meiotic error and chromosome length, we fit a logistic GLM with the response variable encoded as counts of BPH and non-BPH blastomeres for each chromosome for cases stratified into maternal age groups (rounding to the nearest year). Predictor variables included maternal age, chromosome length, and an interaction of age and chromosome length. In order to model overdispersion, we did not fix the dispersion parameter (i.e., quasi-binomial).

To calculate relative difference in rates of whole-chromosome abnormalities for the two different sample types, we stratified errors by the total number of affected chromosomes, then used the formula: (*p* − *q*)/*p*, where *p* is the proportion of affected blastomeres and *q* is the proportion of affected TE biopsies with a given number of whole-chromosome abnormalities out of the total sample.

### Data availability

The Stanford University Research Compliance Office deemed this work to not meet the Federal definition of human subjects research, and it was thus exempted from IRB review. This determination was based on the facts that 1) the work involved no intervention or interaction with study subjects, 2) researchers did not obtain or receive individually identifiable private information, and 3) the data or specimens were collected for purposes other than the current research, the identifiers for the data or specimens were replaced with a code, and the research team was prohibited from obtaining the key to the code. Natera, Inc. also received an IRB exemption for this retrospective examination of the de-identified prenatal genetic screening data in a review conducted by Ethical & Independent Review Services. Analysis code and auxiliary files are available via GitHub: https://github.com/rmccoy7541/aneuploidy_analysis. De-identified primary data are shared with [37], and ploidy calls are available in supplemental materials of that publication. Questions regarding the detection of aneuploidy and the underlying genotype data should be addressed to Zachary Demko (zdemko@natera.com).

## Supporting Information

S1 Fig Maternal age association with whole-chromosome abnormalities is chromosome-dependent. Proportion of affected blastomeres versus maternal age, stratified by chromosome. Error bars indicate standard errors of the proportions. The final panel overlays data from all chromosomes for the purpose of comparison.
(TIFF)

S2 Fig Decline in proportion of euploid blastomeres with maternal age is strongly concordant with decline in IVF success rates with maternal age. **A**: Rates of day-3 blastomere euploidy (requiring no detected segmental errors) versus maternal age compared to IVF success rates. Per-cycle IVF success rate data are from the 2011 CDC National Summary Report [34], while ploidy data are from our study. Error bars (included only for the ploidy data series) indicate standard errors of the proportions. Age groups including fewer than 10 embryos were not plotted to improve figure clarity. **B**: Rates of whole-chromosome abnormalities versus maternal age for egg donors (*n* = 535) and non-donor patients (*n* = 2,374). Maternal age refers to the age of the individual from whom the oocyte was obtained (i.e. the egg donor or the non-donor patient). Controlling for maternal age, rates of whole-chromosome abnormalities were not different between these groups (*β* = 0.0757, *SE* = 0.0707, *P* = 0.284).
(TIFF)

S3 Fig The maternal age effect on rate of whole-chromosome abnormalities is chromosome specific, with a bias of maternal meiotic errors toward smaller chromosomes. **A**: Chromosome-specific rates of error for mothers less than and greater than or equal to 35 years of age. Deviations from the x = y line indicate age effects on error incidence, with the steep slope in the data reflecting an interaction between the effects of maternal age and chromosome length on BPH error (*P* = 1.00 × 10^−9^). Error bars indicate standard errors of the proportions. **B**: Coefficient estimates (± standard error) of a logistic regression model testing for an association between rate of whole-chromosome abnormalities of specific chromosomes and maternal age.
(TIFF)

S4 Fig Rates of various forms of whole-chromosome abnormality with respect to maternal age, stratified by sample type. Error bars indicate standard errors of the proportions. Age groups including fewer than 10 embryos were excluded to improve figure clarity. maternal trisomy (*β* = 0.0785, *SE* = 0.00322, *P* < 1 × 10^−10^), maternal monosomy (*β* = 0.0765, *SE* = 0.00307, *P* < 1 × 10^−10^), maternal uniparental disomy (*β* = 0.0377, *SE* = 0.00877, *P* = 1.75 × 10^−5^), and nullisomy (*β* = 0.0204, *SE* = 0.00429, *P* = 2.15 × 10^−6^) all significantly increased with maternal age in blastomere samples. The same forms of error also increased with maternal age in TE biopsies (maternal trisomy: *β* = 0.110, *SE* = 5.024 × 10^−3^, *P* < 1 × 10^−10^; maternal monosomy: *β* = 0.120, *SE* = 0.00535, *P* < 1 × 10^−10^; nullisomy: *β* = 0.0404, *SE* = 0.0123, *P* = 1.01 × 10^−3^), with the exception of maternal uniparental disomy, which is rare at day 5 (*β* = 0.0386, *SE* = 0.0255, *P* = 0.129).
(TIFF)

S1 Table Best-fit generalized linear models describing the relationship between probability of whole-chromosome abnormalities and maternal age, stratified by sample type. Dispersion parameter for quasibinomial family taken to be 1.348 for the blastomere model and 1.280 for the TE biopsy model.
(PDF)

S2 Table Generalized linear model results describing the relationship between probability of maternal BPH trisomy per-chromosome with relation to maternal age and chromosome length. Dispersion parameter for quasibinomial family taken to be 2.662.
(PDF)

S3 Table Associations between referral reasons and incidence of whole-chromosome abnormalities: day-3 blastomeres. Full generalized linear model results, where the dependent variable is counts of biopsies inferred to be euploid or non-euploid. Dispersion parameter for quasibinomial family taken to be 1.333.
(PDF)

S4 Table Associations between referral reasons and incidence of whole-chromosome abnormalities: day-5 TE biopsies. Full generalized linear model results, where the dependent variable is counts of biopsies inferred to be euploid or non-euploid. Dispersion parameter for quasibinomial family taken to be 1.258.
(PDF)

S5 Table Associations between referral reasons and meiotic error: day-3 blastomeres. Full generalized linear model results, where the dependent variable is counts of biopsies inferred to contain a BPH error versus those that do not. Dispersion parameter for quasibinomial family taken to be 1.253.
(PDF)

S6 Table Associations between referral reasons and meiotic error: day-5 TE biopsies. Full generalized linear model results, where the dependent variable is counts of biopsies inferred to contain a BPH error versus those that do not. Dispersion parameter for quasibinomial family taken to be 1.0943.
(PDF)

S7 Table Associations between referral reasons and mitotic error: day-3 blastomeres. Full generalized linear model results, where the dependent variable is counts of biopsies inferred to contain a paternal chromosome error versus those that do not. Dispersion parameter for quasibinomial family taken to be 1.413.
(PDF)

S8 Table Associations between referral reasons and mitotic error: day-5 TE biopsies. Full generalized linear model results, where the dependent variable is counts of biopsies inferred to contain a paternal chromosome error versus those that do not. Dispersion parameter for quasibinomial family taken to be 1.280.
(PDF)
