# Supplementary figures and images for: Evidence of Selection against Complex Mitotic-Origin Aneuploidy during Preimplantation Development
Source: PLoS Genet. 2015 Oct 22;11(10):e1005601. doi: 10.1371/journal.pgen.1005601 (PMC4619652; doi:10.1371/journal.pgen.1005601)

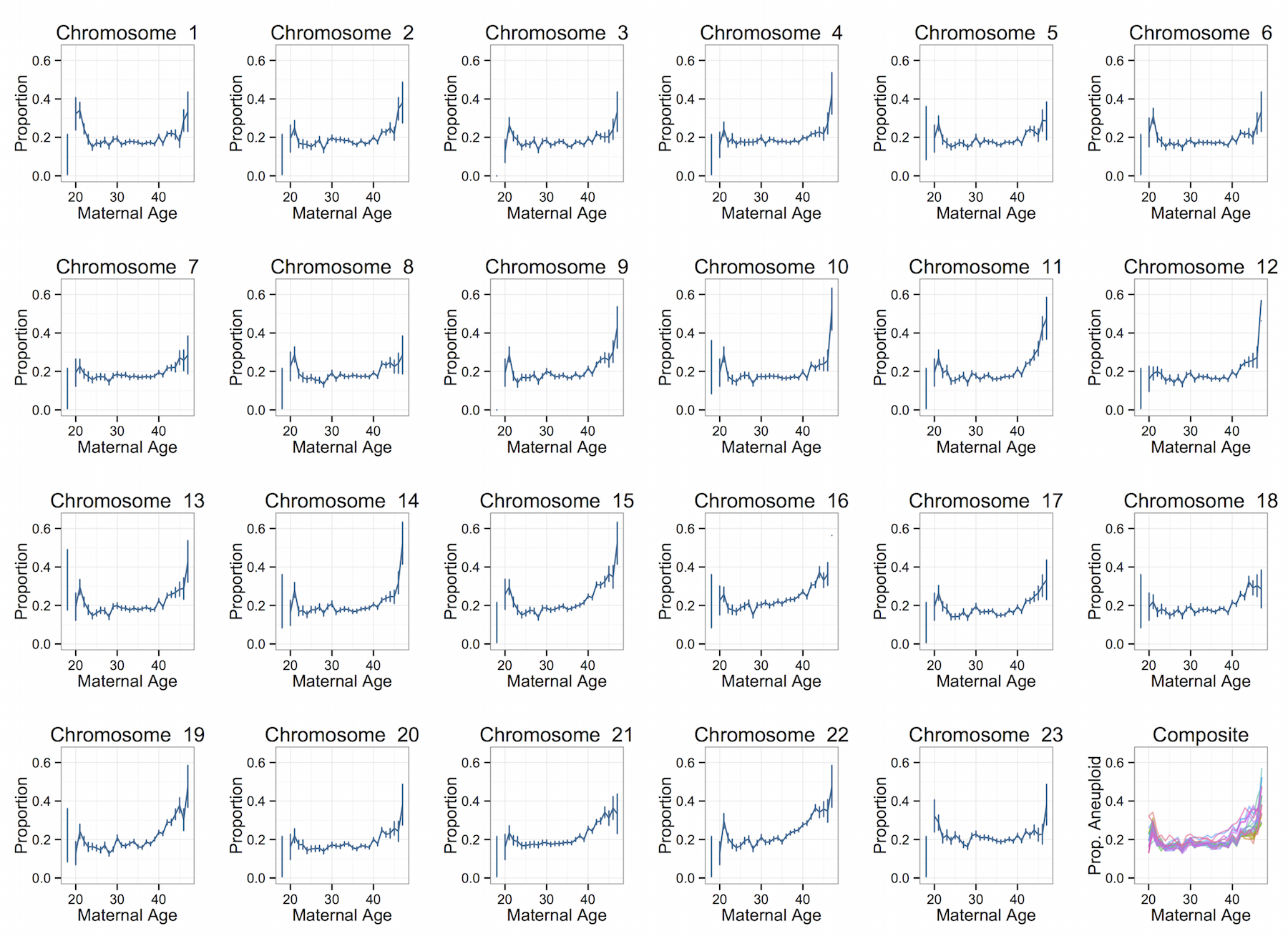

Supplement: S1 Fig — Proportion of affected blastomeres versus maternal age, stratified by chromosome. Error bars indicate standard errors of the proportions. The final panel overlays data from all chromosomes for the purpose of comparison. (TIFF) [file pgen.1005601.s001.tiff]

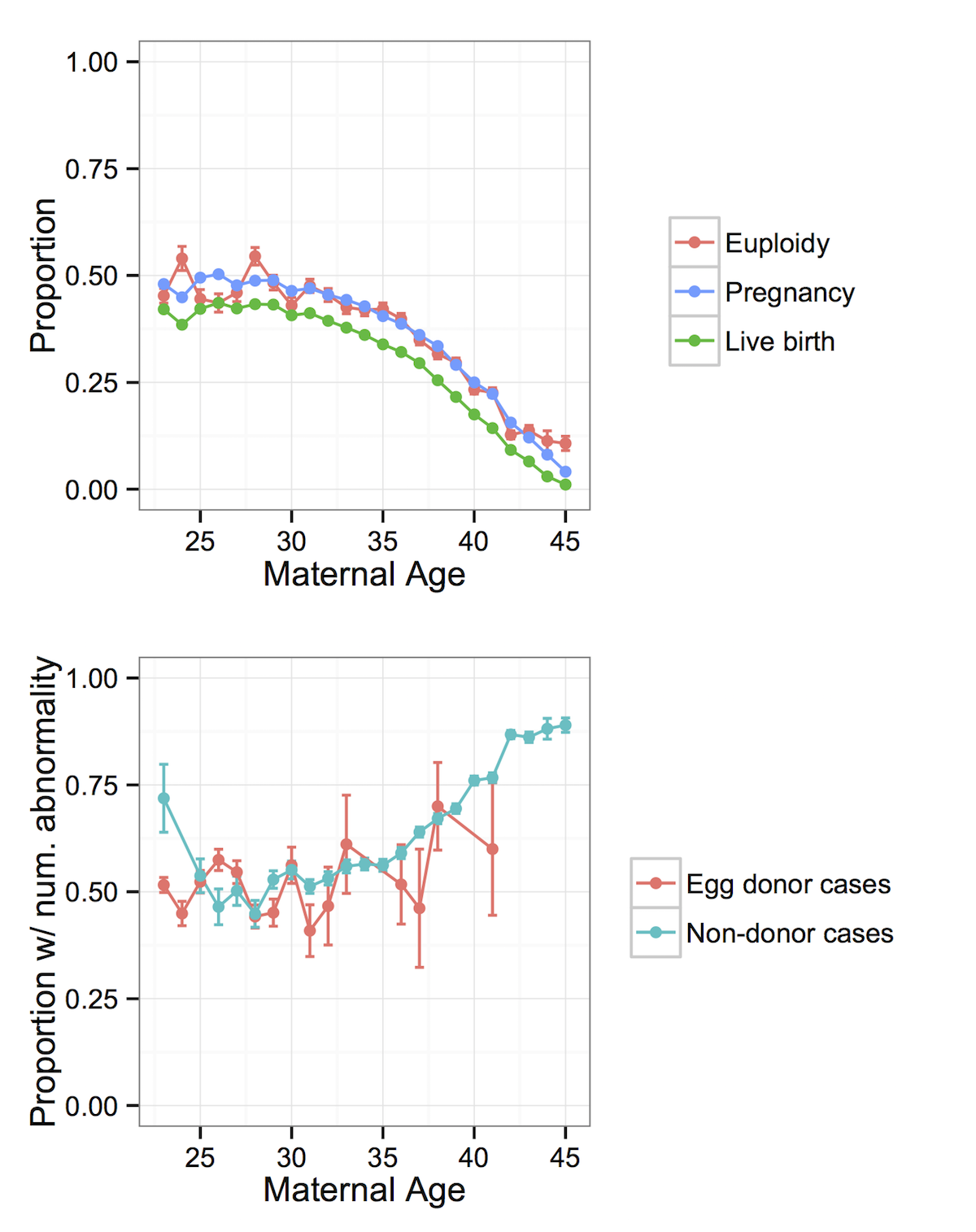

Supplement: S2 Fig — A: Rates of day-3 blastomere euploidy (requiring no detected segmental errors) versus maternal age compared to IVF success rates. Per-cycle IVF success rate data are from the 2011 CDC National Summary Report [34], while ploidy data are from our study. Error bars (included only for the ploidy data series) indicate standard errors of the proportions. Age groups including fewer than 10 embryos were not plotted to improve figure clarity. B: Rates of whole-chromosome abnormalities versus maternal age for egg donors (n = 535) and non-donor patients (n = 2,374). Maternal age refers to the age of the individual from whom the oocyte was obtained (i.e. the egg donor or the non-donor patient). Controlling for maternal age, rates of whole-chromosome abnormalities were not different between these groups (β = 0.0757, SE = 0.0707, P = 0.284). (TIFF) [file pgen.1005601.s002.tiff]

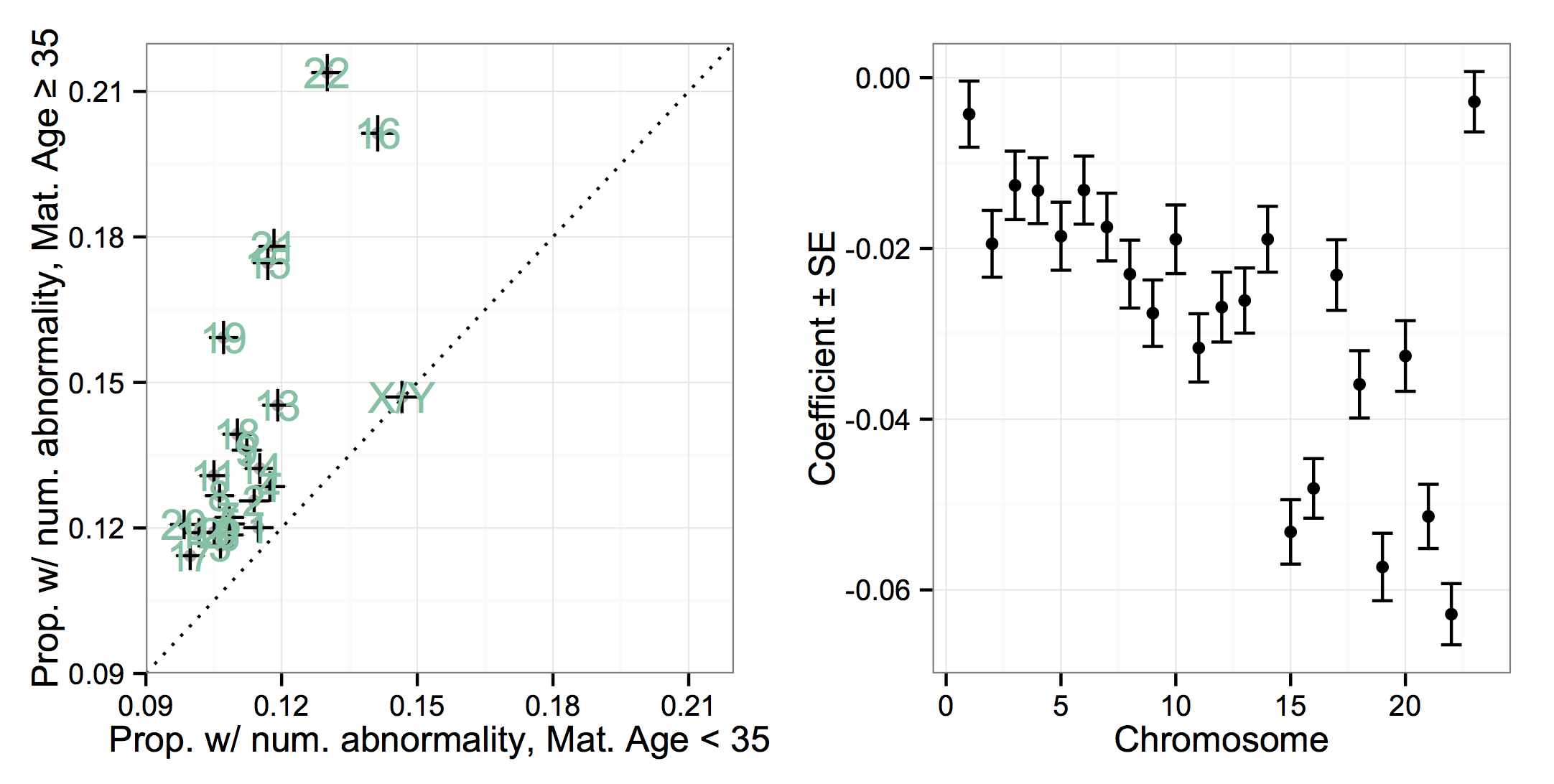

Supplement: S3 Fig — A: Chromosome-specific rates of error for mothers less than and greater than or equal to 35 years of age. Deviations from the x = y line indicate age effects on error incidence, with the steep slope in the data reflecting an interaction between the effects of maternal age and chromosome length on BPH error (P = 1.00 × 10^−9^). Error bars indicate standard errors of the proportions. B: Coefficient estimates (± standard error) of a logistic regression model testing for an association between rate of whole-chromosome abnormalities of specific chromosomes and maternal age. (TIFF) [file pgen.1005601.s003.tiff]

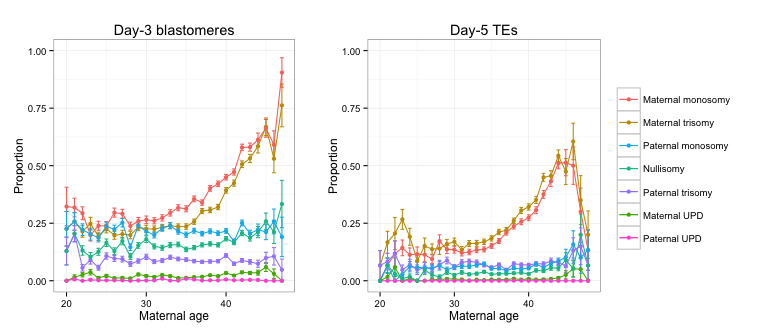

Supplement: S4 Fig — Error bars indicate standard errors of the proportions. Age groups including fewer than 10 embryos were excluded to improve figure clarity. maternal trisomy (β = 0.0785, SE = 0.00322, P < 1 × 10^−10^), maternal monosomy (β = 0.0765, SE = 0.00307, P < 1 × 10^−10^), maternal uniparental disomy (β = 0.0377, SE = 0.00877, P = 1.75 × 10^−5^), and nullisomy (β = 0.0204, SE = 0.00429, P = 2.15 × 10^−6^) all significantly increased with maternal age in blastomere samples. The same forms of error also increased with maternal age in TE biopsies (maternal trisomy: β = 0.110, SE = 5.024 × 10^−3^, P < 1 × 10^−10^; maternal monosomy: β = 0.120, SE = 0.00535, P < 1 × 10^−10^; nullisomy: β = 0.0404, SE = 0.0123, P = 1.01 × 10^−3^), with the exception of maternal uniparental disomy, which is rare at day 5 (β = 0.0386, SE = 0.0255, P = 0.129). (TIFF) [file pgen.1005601.s004.tiff]
