# Supplementary material for: Evidence of Selection against Complex Mitotic-Origin Aneuploidy during Preimplantation Development
Source: PLoS Genet. 2015 Oct 22;11(10):e1005601. doi: 10.1371/journal.pgen.1005601 (PMC4619652; doi:10.1371/journal.pgen.1005601)
Supplement: S1 Table — Dispersion parameter for quasibinomial family taken to be 1.348 for the blastomere model and 1.280 for the TE biopsy model. (PDF) [file pgen.1005601.s005.pdf]

S1 Table. Best-fit generalized linear models describing the relationship between probability of whole-chromosome abnormalities and maternal age, stratified by sample type. Dispersion parameter for quasibinomial family taken to be 1.348 for the blastomere model and 1.280 for the TE biopsy model.

**Day-3 blastomeres**				
Variable	β	SE	t	P
(Intercept)	-1.246	2.713	-0.459	0.646
Maternal age	0.267	0.257	1.042	0.297
(Maternal age)^2	-0.0143	7.941×10^{-3}	-1.799	0.0721
(Maternal age)^3	2.265×10^{-4}	8.062×10^{-5}	2.809	0.0050
**Day-5 TE biopsies**				
Variable	β	SE	t	P
(Intercept)	4.846	0.776	6.239	4.98×10^{-10}
Maternal age	-0.416	0.0450	-9.242	$< 1 \times 10^{-10}$
(Maternal age)^2	7.550×10^{-3}	6.468×10^{-4}	11.672	$< 1 \times 10^{-10}$
