# Supplementary material for: Evidence of Selection against Complex Mitotic-Origin Aneuploidy during Preimplantation Development
Source: PLoS Genet. 2015 Oct 22;11(10):e1005601. doi: 10.1371/journal.pgen.1005601 (PMC4619652; doi:10.1371/journal.pgen.1005601)
Supplement: S2 Table — Dispersion parameter for quasibinomial family taken to be 2.662. (PDF) [file pgen.1005601.s006.pdf]

S2 Table. Generalized linear model results describing the relationship between probability of maternal BPH trisomy per-chromosome with relation to maternal age and chromosome length. Dispersion parameter for quasibinomial family taken to be 2.662.

Variable	β	SE	t	P
(Intercept)	0.773	0.736	1.049	0.294
Chromosome length	-4.473×10^{-9}	2.417×10^{-9}	-1.851	0.0647
Maternal age	0.294	0.0392	7.487	$< 1 \times 10^{-10}$
(Maternal age)^2	-5.907×10^{-3}	5.392×10^{-4}	-10.955	$< 1 \times 10^{-10}$
Chrom. length $\times$ Maternal age	2.494×10^{-10}	6.423×10^{-11}	3.882	1.15×10^{-4}
