# Supplementary material for: Evidence of Selection against Complex Mitotic-Origin Aneuploidy during Preimplantation Development
Source: PLoS Genet. 2015 Oct 22;11(10):e1005601. doi: 10.1371/journal.pgen.1005601 (PMC4619652; doi:10.1371/journal.pgen.1005601)
Supplement: S3 Table — Full generalized linear model results, where the dependent variable is counts of biopsies inferred to be euploid or non-euploid. Dispersion parameter for quasibinomial family taken to be 1.333. (PDF) [file pgen.1005601.s007.pdf]

S3 Table. Associations between referral reasons and incidence of whole-chromosome abnormalities: day-3 blastomeres. Full generalized linear model results, where the dependent variable is counts of biopsies inferred to be euploid or non-euploid. Dispersion parameter for quasibinomial family taken to be 1.333.

Variable	β	SE	t	P
(Intercept)	8.387	1.382	6.066	1.74×10^{-9}
Maternal age	−0.571	0.0793	−7.205	$< 1 \times 10^{-10}$
(Maternal age)^2	0.00969	0.00113	8.590	$< 1 \times 10^{-10}$
Recurrent pregnancy loss	−0.00945	0.0630	−0.150	0.881
Previous IVF failure	0.181	0.0752	2.413	0.0160
Male factor	0.140	0.112	1.259	0.208
Unexplained infertility	0.0176	0.109	0.162	0.871
Translocation carrier	0.438	0.123	3.563	0.00038
Previous aneuploidy	0.0944	0.0899	−1.051	0.294
