# Supplementary material for: Evidence of Selection against Complex Mitotic-Origin Aneuploidy during Preimplantation Development
Source: PLoS Genet. 2015 Oct 22;11(10):e1005601. doi: 10.1371/journal.pgen.1005601 (PMC4619652; doi:10.1371/journal.pgen.1005601)
Supplement: S4 Table — Full generalized linear model results, where the dependent variable is counts of biopsies inferred to be euploid or non-euploid. Dispersion parameter for quasibinomial family taken to be 1.258. (PDF) [file pgen.1005601.s008.pdf]

S4 Table. Associations between referral reasons and incidence of whole-chromosome abnormalities: day-5 TE biopsies. Full generalized linear model results, where the dependent variable is counts of biopsies inferred to be euploid or non-euploid. Dispersion parameter for quasibinomial family taken to be 1.258.

Variable	β	SE	t	P
(Intercept)	4.690	1.074	4.366	1.32×10^{-5}
Maternal age	−0.414	0.0609	−6.803	$< 1 \times 10^{-10}$
(Maternal age)^2	0.00755	0.000856	8.814	$< 1 \times 10^{-10}$
Recurrent pregnancy loss	0.131	0.0494	2.641	0.00831
Previous IVF failure	0.138	0.0585	2.352	0.0188
Male factor	−0.0252	0.0764	−0.330	0.741
Unexplained infertility	−0.0469	0.0691	−0.680	0.497
Translocation carrier	0.502	0.119	4.217	2.57×10^{-5}
Previous aneuploidy	0.0573	0.0786	0.729	0.466
