# Supplementary material for: Evidence of Selection against Complex Mitotic-Origin Aneuploidy during Preimplantation Development
Source: PLoS Genet. 2015 Oct 22;11(10):e1005601. doi: 10.1371/journal.pgen.1005601 (PMC4619652; doi:10.1371/journal.pgen.1005601)
Supplement: S5 Table — Full generalized linear model results, where the dependent variable is counts of biopsies inferred to contain a BPH error versus those that do not. Dispersion parameter for quasibinomial family taken to be 1.253. (PDF) [file pgen.1005601.s009.pdf]

S5 Table. Associations between referral reasons and meiotic error: day-3 blastomeres. Full generalized linear model results, where the dependent variable is counts of biopsies inferred to contain a BPH error versus those that do not. Dispersion parameter for quasibinomial family taken to be 1.253.

Variable	β	SE	t	P
(Intercept)	1.756	1.567	1.121	0.263
Maternal age	−0.306	0.0867	−3.531	0.000 429
(Maternal age)^2	0.00601	0.00119	5.047	5.16×10^{-7}
Recurrent pregnancy loss	0.0175	0.0684	0.256	0.798
Previous IVF failure	0.0656	0.0773	0.849	0.396
Male factor	−0.116	0.130	−0.896	0.370
Unexplained infertility	0.214	0.123	1.741	0.0819
Translocation carrier	0.743	0.127	5.844	6.54×10^{-9}
Previous aneuploidy	−0.0113	0.0922	0.123	0.902
