# Supplementary material for: Evidence of Selection against Complex Mitotic-Origin Aneuploidy during Preimplantation Development
Source: PLoS Genet. 2015 Oct 22;11(10):e1005601. doi: 10.1371/journal.pgen.1005601 (PMC4619652; doi:10.1371/journal.pgen.1005601)
Supplement: S6 Table — Full generalized linear model results, where the dependent variable is counts of biopsies inferred to contain a BPH error versus those that do not. Dispersion parameter for quasibinomial family taken to be 1.0943. (PDF) [file pgen.1005601.s010.pdf]

S6 Table. Associations between referral reasons and meiotic error: day-5 TE biopsies. Full generalized linear model results, where the dependent variable is counts of biopsies inferred to contain a BPH error versus those that do not. Dispersion parameter for quasibinomial family taken to be 1.0943.

Variable	β	SE	t	P
(Intercept)	−32.34	25.30	−1.278	0.201
Maternal age	4.731	3.076	1.538	0.124
(Maternal age)^2	0.259	0.138	−1.882	0.0600
(Maternal age)^3	5.929×10^{-3}	2.699×10^{-3}	2.197	0.0281
(Maternal age)^4	4.800×10^{-5}	1.956×10^{-5}	−2.455	0.0142
Recurrent pregnancy loss	0.0127	0.0584	2.172	0.0299
Previous IVF failure	0.00934	0.0701	1.332	0.183
Male factor	−0.181	0.107	−1.695	0.0902
Unexplained infertility	0.0425	0.0851	0.499	0.618
Translocation carrier	0.366	0.150	2.445	0.0146
Previous aneuploidy	−0.0118	0.0911	−0.130	0.898
