# Supplementary material for: Evidence of Selection against Complex Mitotic-Origin Aneuploidy during Preimplantation Development
Source: PLoS Genet. 2015 Oct 22;11(10):e1005601. doi: 10.1371/journal.pgen.1005601 (PMC4619652; doi:10.1371/journal.pgen.1005601)
Supplement: S7 Table — Full generalized linear model results, where the dependent variable is counts of biopsies inferred to contain a paternal chromosome error versus those that do not. Dispersion parameter for quasibinomial family taken to be 1.413. (PDF) [file pgen.1005601.s011.pdf]

S7 Table. Associations between referral reasons and mitotic error: day-3 blastomeres. Full generalized linear model results, where the dependent variable is counts of biopsies inferred to contain a paternal chromosome error versus those that do not. Dispersion parameter for quasibinomial family taken to be 1.413.

Variable	β	SE	t	P
(Intercept)	−1.008	0.0349	−28.83	$< 1 \times 10^{-10}$
Recurrent pregnancy loss	0.0842	0.0667	1.264	0.207
Previous IVF failure	0.191	0.0756	2.534	0.0114
Male factor	0.141	0.117	1.205	0.229
Unexplained infertility	−0.189	0.125	−1.512	0.131
Translocation carrier	−0.0285	0.129	−0.220	0.826
Previous aneuploidy	−0.183	0.0967	−1.889	0.0591
