# Supplementary material for: Evidence of Selection against Complex Mitotic-Origin Aneuploidy during Preimplantation Development
Source: PLoS Genet. 2015 Oct 22;11(10):e1005601. doi: 10.1371/journal.pgen.1005601 (PMC4619652; doi:10.1371/journal.pgen.1005601)
Supplement: S8 Table — Full generalized linear model results, where the dependent variable is counts of biopsies inferred to contain a paternal chromosome error versus those that do not. Dispersion parameter for quasibinomial family taken to be 1.280. (PDF) [file pgen.1005601.s012.pdf]

S8 Table. Associations between referral reasons and mitotic error: day-5 TE biopsies. Full generalized linear model results, where the dependent variable is counts of biopsies inferred to contain a paternal chromosome error versus those that do not. Dispersion parameter for quasibinomial family taken to be 1.280.

Variable	β	SE	t	P
(Intercept)	−2.107	0.0494	−42.65	$< 1 \times 10^{-10}$
Recurrent pregnancy loss	0.120	0.0728	1.645	0.100
Previous IVF failure	0.213	0.0830	2.565	0.0104
Male factor	−0.0625	0.115	−0.544	0.587
Unexplained infertility	−0.0741	0.107	−0.693	0.488
Translocation carrier	0.135	0.172	0.781	0.435
Previous aneuploidy	0.0818	0.114	0.720	0.472
